# A Novel Overall Survival Prediction Signature Based on Comprehensive Research in Prostate Cancer Bone Metastases

**DOI:** 10.3389/fmed.2022.815541

**Published:** 2022-06-16

**Authors:** Konghe Hu, Xinyue Hu, Yang Duan, Wenqiang Li, Jing Qian, Junjie Chen

**Affiliations:** ^1^Department of Spine Surgery, The Affiliated Yuebei People's Hospital of Shantou University Medical College, Shaoguan, China; ^2^Department of Clinical Laboratory, Kunming First People's Hospital, Kunming Medical University, Kunming, China; ^3^Department of Spine Surgery, Zhujiang Hospital, Southern Medical University, Guangzhou, China; ^4^Department of Critical Care Medicine, Zhujiang Hospital, Southern Medical University, Guangzhou, China

**Keywords:** differentially expressed genes, prostate adenocarcinoma, diagnostic value, bone metastasis, gene ontology, prostate cancer prognosis

## Abstract

**Background:**

Prostate adenocarcinoma (PRAD)-related bone metastases are a leading source of morbidity and mortality; however, good diagnostic biomarkers are not known yet. The aim of this study was to identify biomarkers and prognostic indicators for the diagnosis and treatment of PRAD-associated bone metastases.

**Methods:**

By combining the data from The Cancer Genome Atlas(TCGA) and PRAD SU2C 2019, We performed a comprehensive analysis of the expression differences, biological functions, and interactions of genes associated with PRAD bone metastasis. Annotation, visualization, and integrated discovery were accomplished through the use of gene ontology enrichment and gene set enrichment analysis. The protein-protein interaction network was constructed using the STRING database, and the diagnostic value of prognostic genes was validated using receiver-operating-characteristic and Kaplan-Meier curves.

**Results:**

Six genes (*DDX47, PRL17, AS3MT, KLRK1, ISLR*, and *S100A8*) associated with PRAD bone metastases were identified; these had prognostic value as well. Among them, enrichment was observed for the biological processes extracellular matrix tissue, extracellular structural tissue, steroid hormone response, and cell oxidative detoxification. KEGG analysis revealed enrichment in interactions with extracellular matrix receptors, diseases including Parkinson's disease and dilated cardiomyopathy, and estrogen signaling pathways. The area under the curve values of 0.8938, 0.9885, and 0.979, obtained from time-dependent receiver-operating-characteristic curve analysis for 1, 3, and 5-year overall survival confirmed the good performance of the model under consideration. S100A8 expression was not detected in the normal prostate tissue but was detected in PRAD.

**Conclusions:**

We identified ISLR as a potential biomarker for PRAD bone metastasis. Moreover, the genes identified to have prognostic value may act as therapeutic targets for PRAD bone metastasis.

## Introduction

Prostate adenocarcinoma (PRAD) is one of the top causes of cancer-related death among male cancer patients. A typical symptom of advanced PRAD is the presence of bone metastasis (1), which is often detected at autopsy in 90% of men who die from the disease (2). Among the patients with metastatic PRAD, 42.9% have bone metastases (3). Globally, the incidence of prostate cancer has risen by 169.11% since 1990, with the majority of the increase occurring in men over the age of 50 (4). The mechanisms that lead to bone metastases and eventually aggravate PRAD is not well understood, even though these are leading causes of morbidity and mortality in individuals with advanced PRAD (5). Taken together, bone metastases from prostate cancer are a challenging clinical problem that needs to be addressed. Therefore, to improve the prognosis, identifying potential target molecules and clarifying the underlying mechanisms of PRAD bone metastasis is of utmost importance.

Many markers of bone metastases in prostate cancer have been reported; for example, RUNX2 is expressed ectopically, indicating that its function is influenced by the tumor microenvironment (6). It was recently discovered that the *TMPRSS2-ERG* gene fusion enhances osteogenic bone metastasis in prostate cancer, implying that particular mutations are responsible for bone metastasis (7). Genes including *FZD8* and *DKK1* have been associated with bone metastases in prostate cancer (8, 9); excessively elevated *UCP1* expressions have been reported as a marker (10). NF-κB has been identified as a major transcription factor in the progression to bone metastases in patients with prostate cancer (11). Cadherin-11 may be a suitable marker for bone metastases progression, which was not expressed in normal prostate epithelial cells, but expressed in prostate cancer, and the expression in lymph nodes, especially in bone, increased gradually from primary lesion to metastatic lesion (12).

Using microarray analysis, it was recently found that several differentially expressed genes (DEGs) and biologically functioning pathways are involved in the development of bone metastases in PRAD (13). Several studies have been carried out to better understand the molecular mechanisms of DEGs identified using microarray analysis. Different microarray types (including gene expression microarray and tissue microarray), tissues [including those of mouse (14) and human (3)], cells (15), and serum prostate specific antigen (16), have been used to conduct genome-wide expression analyses. However, Due to limited sample sizes, use of multiple tissues, and multi-platform analyses, the results of such studies may be inconsistent. Recently, differential gene expression was studied in 38 bone metastatic and 115 non-bone metastatic PRAD tissue and serum samples found that miR-218-5p may be a new serum marker for bone metastasis (17). Furthermore, although numerous bioinformatics analyses have comprehensively studied the known biomarkers, the mechanisms by which functional systems and factors other than biomarkers influence bone metastatic PRAD remain unknown. Thus, there is a need to identify more reliable and accurate markers.

In this study, we used data from TCGA and PRAD SU2C 2019 to analyze significantly DEGs between primary PRAD tissue samples and bone metastatic PRAD using gene ontology (GO) enrichment and gene set enrichment analysis (GSEA) methods to identify the involved biological process (BP). The molecular pathways driving the advancement of PRAD in bone metastases were also investigated using network analysis. Our findings will help to elucidate the molecular mechanisms underlying PRAD bone metastasis at a systems biology level by establishing a complete gene network for this condition.

## Materials and Methods

### Data Preparation

We retrieved a dataset for PRAD (prad_su2c_2019) from Wassim Abida et al. (18) comprising RNA expression profile data, FPKM values of 82 PRAD bone metastasis samples, and 5 *in situ* PRAD tissue samples, survival data, and clinicopathological information of patients, such as gender, and the tissue site. Since the number of PRAD samples was small, we integrated the data from Wassim Abida et al. (18) with TCGA-PRAD data. Gene expression data and FPKM values from RNA sequencing of TCGA-PRAD patients (1 metastatic PRAD sample and 504 *in situ* PRAD tissue samples) were downloaded from UCSC xena (http://xena.ucsc.edu/) (19). This also included survival information and clinicopathological information of patients, such as sex, age, and cancer stage. Then, we randomly selected the same number of samples for *in situ* PRAD and bone metastasis PRAD from the prad_su2c_2019 dataset and TCGA and integrated the expression profile and clinical information. This integrated dataset was divided into a training set (70%) and a validation set (30%).

### Identification of Prognosis-Related Genes and Construction of Prognostic Models

To identify genes for prognosis, we first screened genes closely associated with bone metastases (|logFC| ≥ 2 and adjusted p-value <0.05) for association with PRAD bone metastasis and *in situ* PRAD using the limma package (version 3.48.3) (20). To further assess the impact of bone metastasis-associated gene expression combined with clinicopathological characteristics on disease prognosis, we performed one-way regression analysis for genes associated with bone metastasis (*p* < 0.05). Subsequently, we performed the prognostic analysis of bone metastasis-associated genes, using gene expression quartiles (25% quartiles) as a criterion to classify the samples into high- and low-expression groups. We analyzed the relationship between gene expression and prognosis based on survival information. Moving further, we used these bone metastasis-prognostic genes to construct a multiple regression model and calculated the risk score. For categorizing patients into high- and low-risk categories, the median risk score was utilized as the cutoff point. The Wilcoxon test was applied for comparing and analyzing the statistical significance between two sets of categorical variables.

### Enrichment Analysis and Protein-Protein Interaction Network Construction

To identify differences between the high- and low-risk groups, DEGs were analyzed using the limma package (R software version 3.48.3) (20), with |logFC| ≥ 2 and adjusted *p-*value <0.05 set as thresholds for the differential expression of genes.

Large-scale functional enrichment studies, such as for Biological Process (BP), molecular function (MF), and cellular component (CC), often use the Gene Ontology (GO) analysis approach. Data on genomes, biological pathways, diseases, and medications are stored in Kyoto Encyclopedia of Genes and Genomes (KEGG). GO annotation and KEGG pathway enrichment analyses were performed using clusterProfiler (version 4.0.5) (21) for both high- and low-risk groups of DEGs, and FDR <0.05 was considered statistically significant. The GOplot package (version 1.0.2) (22) was used to visualize the enrichment analysis results.

GSEA is a computational method for analyzing whether a particular gene set is statistically different between two biological states. This method is commonly used to estimate changes in the pathway and BP activity in samples of expression datasets (23). To investigate the differences in BPs between subgroups based on the gene expression profile dataset of PRAD patients, we performed GSEA of Hallmark genes using clusterProfiler (version 4.0.5) (21). The Hallmark gene set (h.all.v7.4.symbols.gmt) was downloaded from MSigDB (https://www.gsea-msigdb.org/gsea/index.jsp) (24) and used for GSEA; corrected *p-*values <0.05 were considered statistically significant. The results were visualized using the enrichplot package (version 1.12.2) (25).

To further analyze the differences in bone metastasis-related pathways in high- and low-risk groups, we specifically focused on the differences in the expression activity of different pathways including angiogenesis, apoptosis, cell cycle, and DNA replication. The Hallmark (angiogenesis and apoptosis) and KEGG (cell cycle and DNA replication) gene sets of different pathways are available at MSigDB (https://www.gsea-msigdb.org/gsea/index.jsp) (24). Gene set variation analysis (GSVA) is a new, non-parametric, unsupervised method that assesses the pathway enrichment of each sample using the given expression dataset. The new GSVA enrichment scoring facilitates the application of functional enrichment in a pathway-centric manner. The R package GSVA (version 1.40.1) (26) was used to assess the differences in the activity of these five pathways in high- and low-risk groups.

STRING (https://string-db.org/) (27), a database of protein-protein interactions (PPIs), contains data on 9.6 million proteins and 13.8 million interactions among proteins. Using this database, PPI networks can be constructed for selected genes. PPI networks help understand the number, type, and extent of interactions proteins can undergo. To explore their interactions, we mapped the DEGs in high- and low-risk groups to the PPI network of the STRING database. To filter out the gene pairs with weak interactions and retain those with strong interactions, the minimum required interaction score was calculated using high confidence setting (0.900); all other parameters were used at default settings. A visual network model was then constructed using Cytoscape (version 3.8.2) (28). The molecular complex detection (MCODE) plugin is used to find key sub-networks and genes in a large network based on the relationships of edges and nodes, facilitating downstream analysis (29). The modules in the network were mined using the MCODE plugin (using default parameters), whereas hub genes were mined using the ClusteringCoefficient algorithm of the CytoHubba plugin (30) using default parameters and selecting the top five genes as hub genes.

### Assessment of the Prognostic Model

To assess the impact of bone metastasis-related prognostic gene expression on prognosis, a risk regression model was constructed by combining clinicopathological characteristics. To assess the independent predictive ability of risk scores on overall survival (OS), the Kaplan-Meier survival curve analysis was performed using the survival package (version 3.2.11) (31). Time-dependent receiver-operating curve (ROC) performance was further evaluated using the R survival ROC package (version 1.0.3) (32) by assessing the area under the curve (AUC) at 1, 3, and 5 years.

To explore the impact of bone metastasis-related prognostic genes on survival, we conducted a risk factor analysis to examine the association between gene expression levels and survival. Subsequently, we considered the ROC curves for each gene to assess the likelihood of the gene being used to predict prognosis. To validate the accuracy of the association of prognostic genes with bone metastasis, we used the validation set to construct a prognostic model based on bone metastasis-related prognostic genes. The dataset was divided into high- and low-risk groups based on risk scores, and Kaplan-Meier survival curve analysis was performed using survival information. Moreover, the AUC of ROC for subjects at 1, 3, and 10 years was evaluated.

### Relationship Between Clinical Factors and Prognosis

To validate the prognostic model of bone metastasis-related genes, we incorporated clinical indicators (markers) into the model and performed univariate and multifactor prognostic analyses using the SURVIVAL package (version 3.2.11) (31). The results are presented as a forest plot. We also performed survival curve analysis after taking clinical factors into consideration. Briefly, data were divided into two groups of prostate and other tissues based on tumor site, and into high- and low-risk groups using the median risk score as a threshold. Survival curve analyses were performed for each group separately. Similarly, data were divided into high- and low-neuroendocrine prostate cancer (NEPC) score groups based on the median scores, and into high- and low-risk groups using the median risk score as the threshold. Survival analysis was then performed for each group.

In addition, we incorporated clinical factors to construct a clinical prediction column line graph (Nomogram). To assess the effect of including clinical factors in the model, we performed decision curve analysis (DCA) using the ggDCA package (version 1.1) (33).

## Results

### Screening of Bone Metastasis Prognosis-Associated Genes

**Figure 1:**
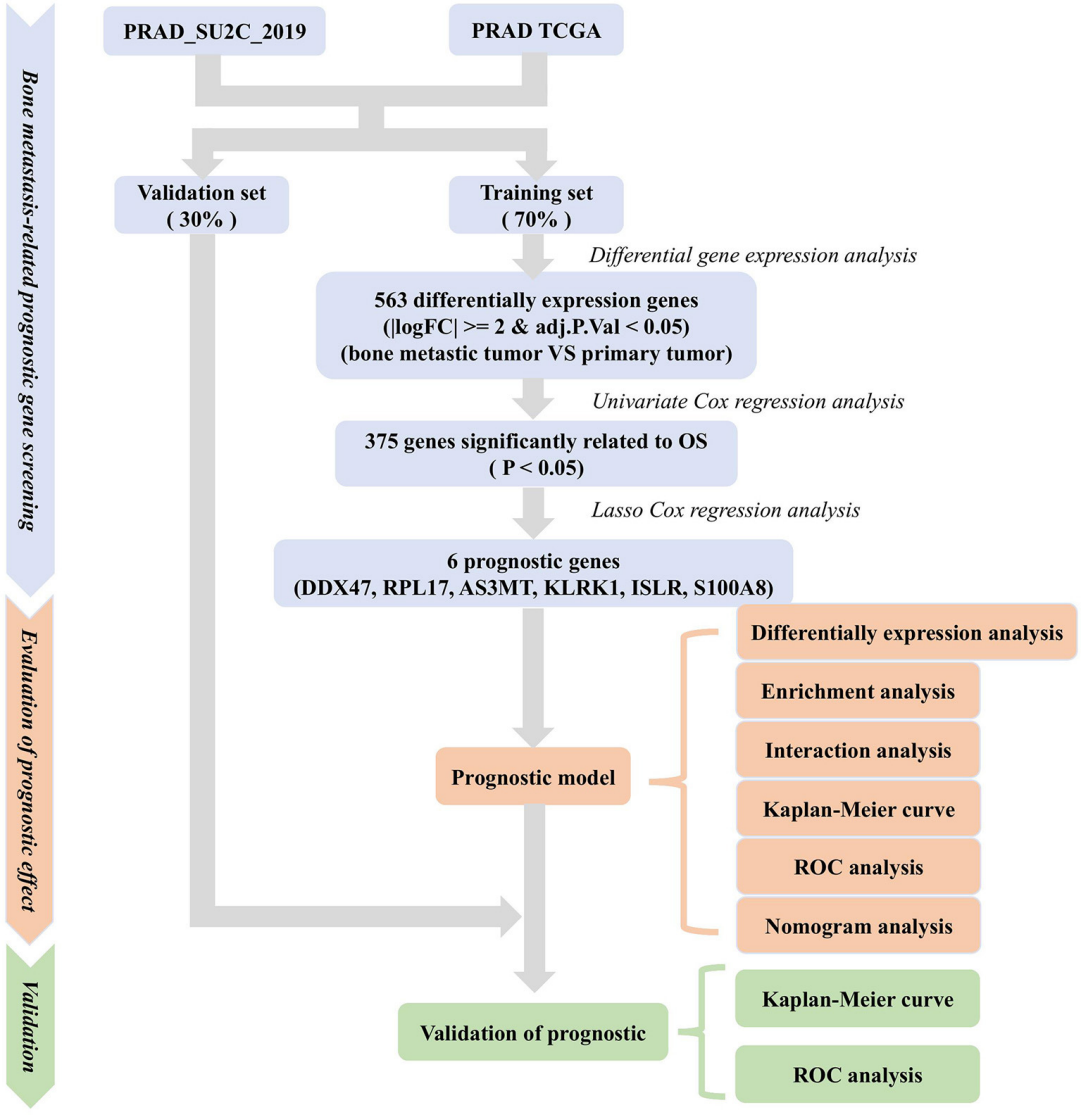
Methodology for screening prognostic genes for bone metastases and constructing prognostic models.

**Figure 2:**
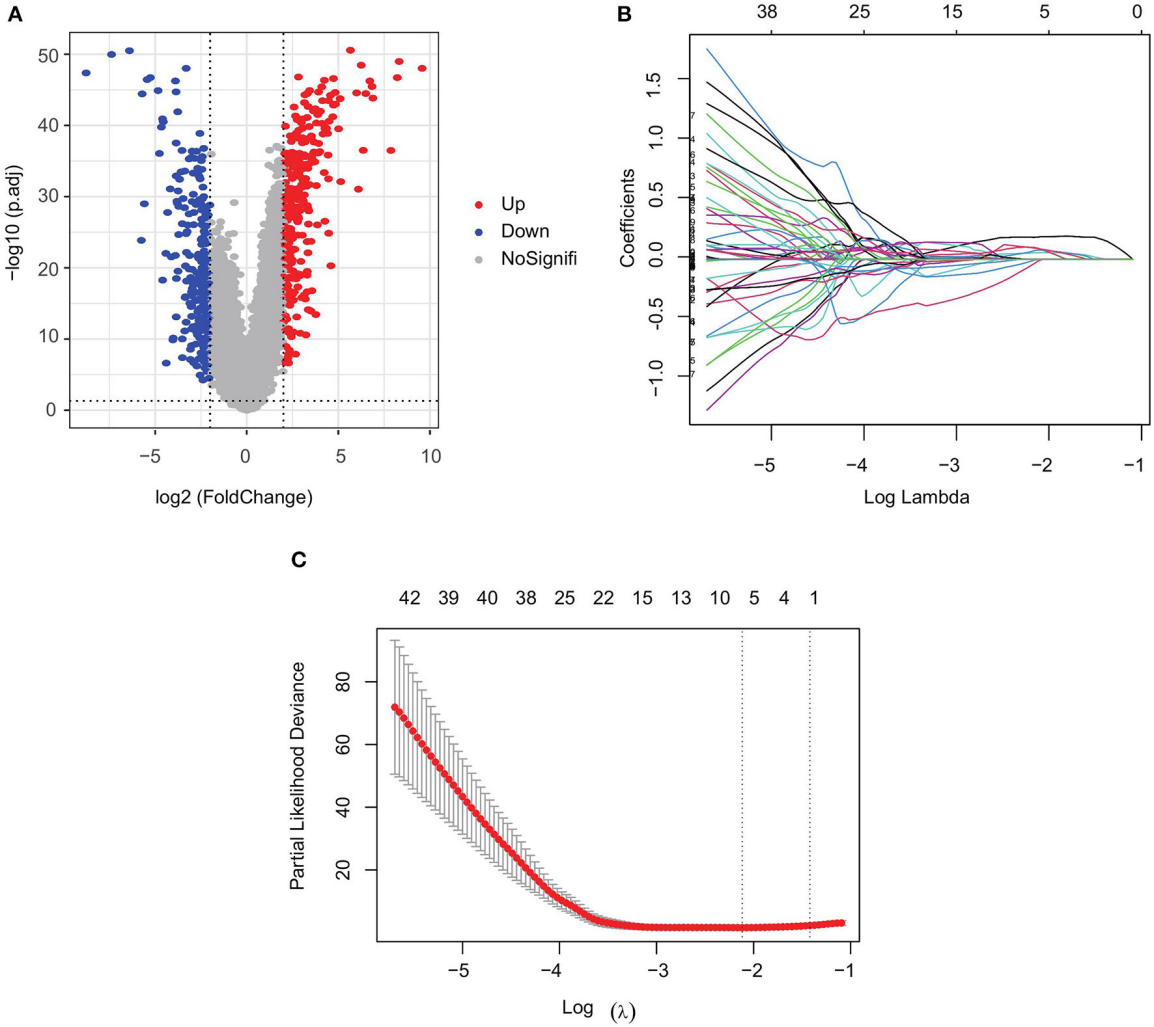
Screening of prognostic genes for bone metastases. **(A)** Differentially expressed genes in PRAD bone metastasis and *in situ* PRAD; **(B)** regression coefficients of each gene in Lasso Cox regression analysis, with certificates representing positive correlations and collaterals representing negative correlations; and **(C)** selection of the best parameter λ in Lasso regression.

We analyzed transcriptomic data from 82 primary PRAD and 82 PRAD bone metastasis samples obtained from TCGA and prad_su2c_2019 to identify prognostic genes. We identified 563 DEGs (Figures 1, 2A) between primary PRAD and bone metastases. Univariate Cox regression analysis yielded 375 bone metastasis genes associated with OS (*p* < 0.05). Screening of these candidate prognostic genes using Lasso Cox regression analysis yielded six prognostic genes (*DDX47, PRL17, AS3MT, KLRK1, ISLR*, and *S100A8*) (Figures 2B,C).

### Construction of Prognostic Model

Using the identified prognostic genes, we constructed a PRAD bone, metastasis-related prognostic model. Multivariate Cox regression analysis was performed for each prognostic gene, and regression coefficients were obtained for each gene. The model was defined as follows: risk score = 0.1317^*^exp(*DDX47*) + 0.0101^*^exp(*RPL17*) + 0.4348^*^exp(*AS3MT*) + 0.0555^*^exp(*KLRK1*) +−0.6110^*^exp(*ISLR*) + 0.2019^*^exp(*S100A8*)). Samples from the training set were evaluated using this risk score formula, and each sample was assigned a risk score and assigned to a risk group.

**Figure 3:**
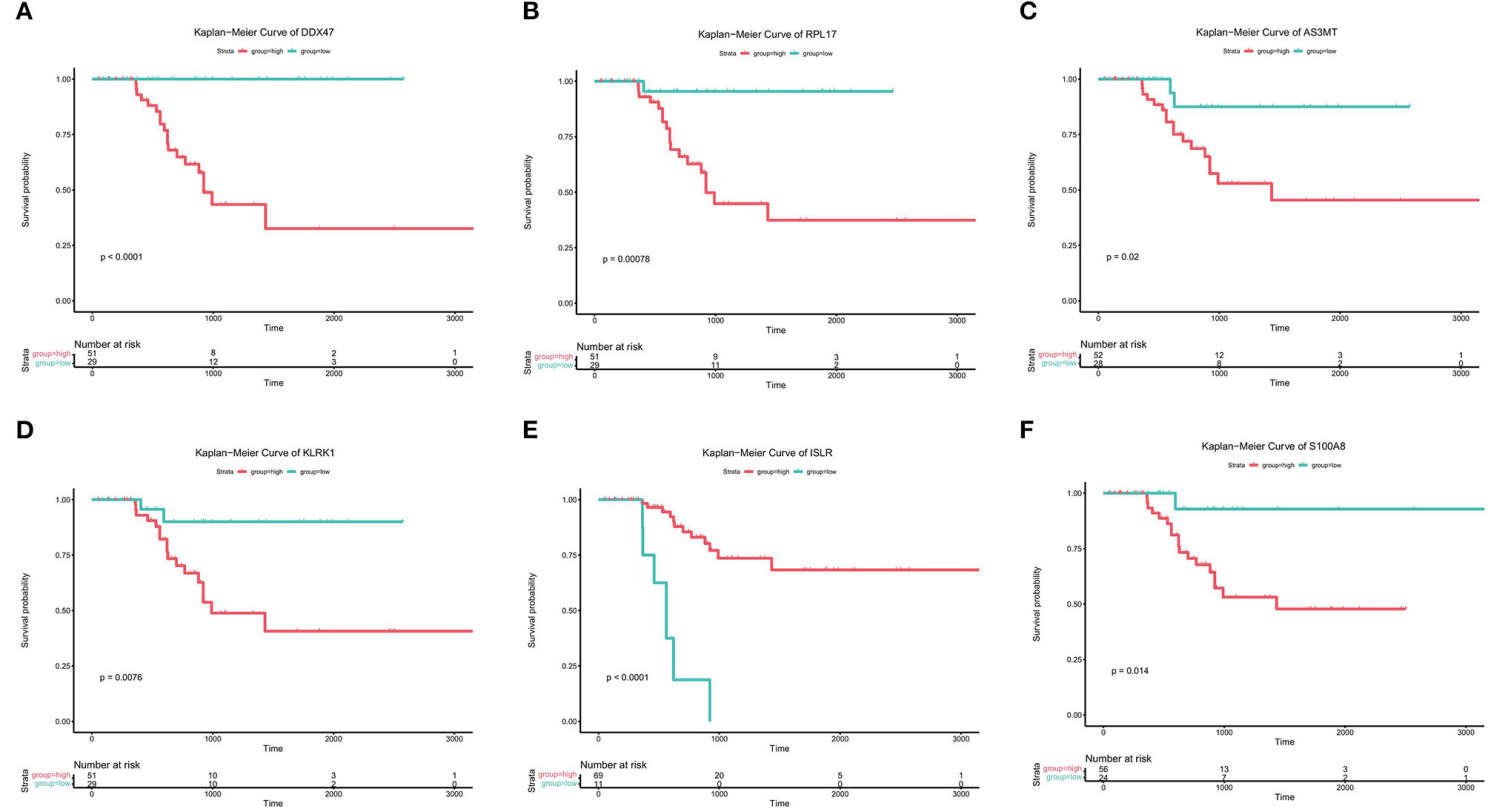
Prognostic analysis of genes associated with bone metastases. **(A-F)** Kaplan-Meier analysis of the survival rates of the high- and low-expression groups of *DDX47, PRPL17, AS3MT, KLRK1, ISLR*, and *S100A8*.

**Figure 4:**
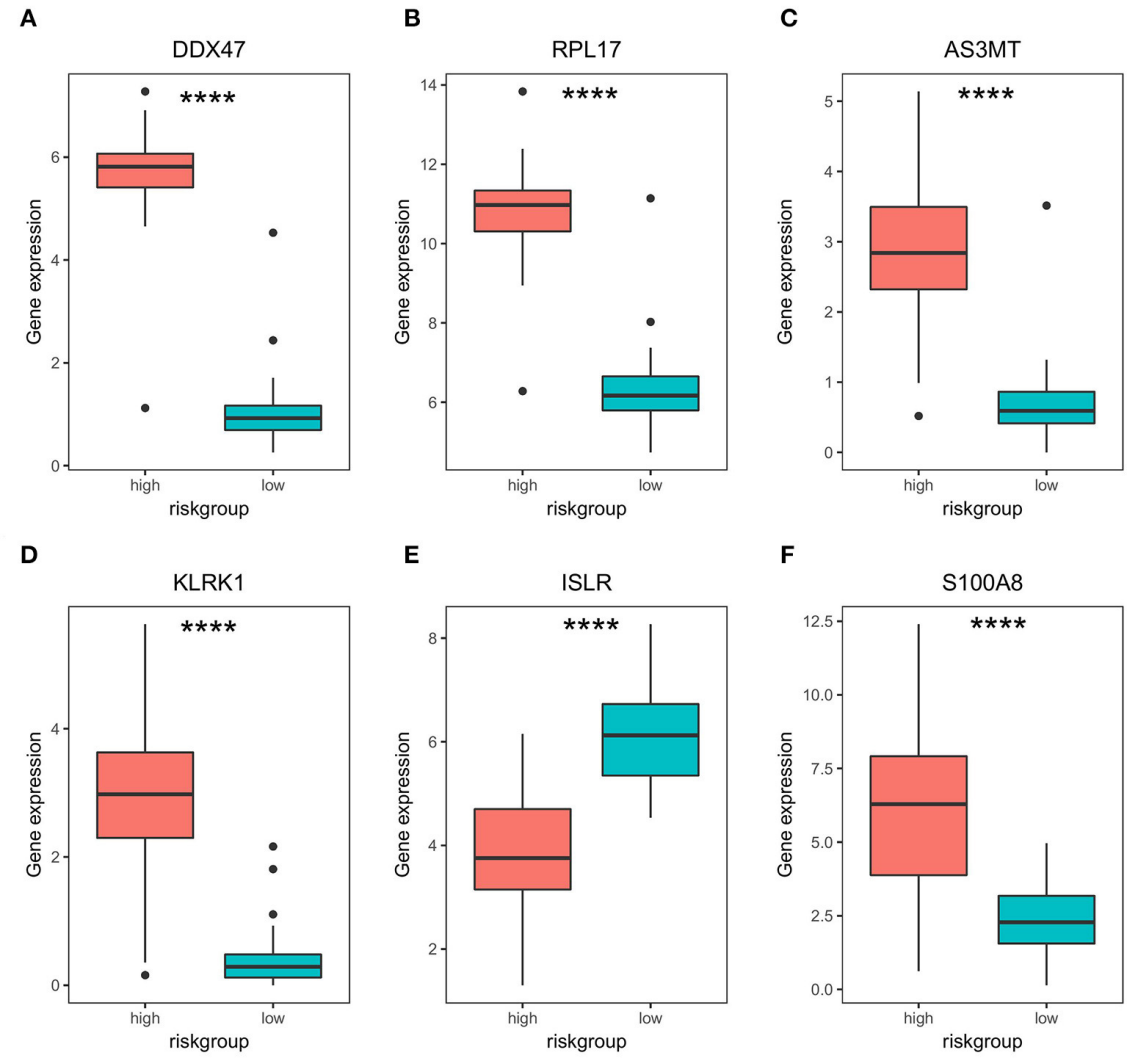
Differential expression of bone metastasis prognostic genes in high- and low-risk groups. **(A–F)** Differential expression analysis of *DDX47, PRPL17, AS3MT, KLRK1, ISLR*, and *S100A8* in high- and low-risk groups. **** represents significant differences, *p* < 0.0001.

Subsequently, to determine the role of each gene in PRAD prognosis, we performed a differential analysis of patient survival by comparing the survival differences between the high- and low-expression groups for each prognostic gene. The results showed that five of the identified prognostic genes (*DDX47, RPL17, AS3MT, KLRK1*, and *S100A8*) were unfavorable factors for patient survival, whereas only one (*ISLR*) was a favorable factor (Figure 3). On assessing the differences in gene expression in both risk groups (Figure 4), we found that the expression of *DDX47, RPL17, AS3MT, KLRK1*, and *S100A8* was significantly higher in the high-risk group than in the low-risk group, and that of *ISLR* was significantly lower in the high-risk group than in the low-risk group.

### GO and KEGG Enrichment Analyses

**Figure 5:**
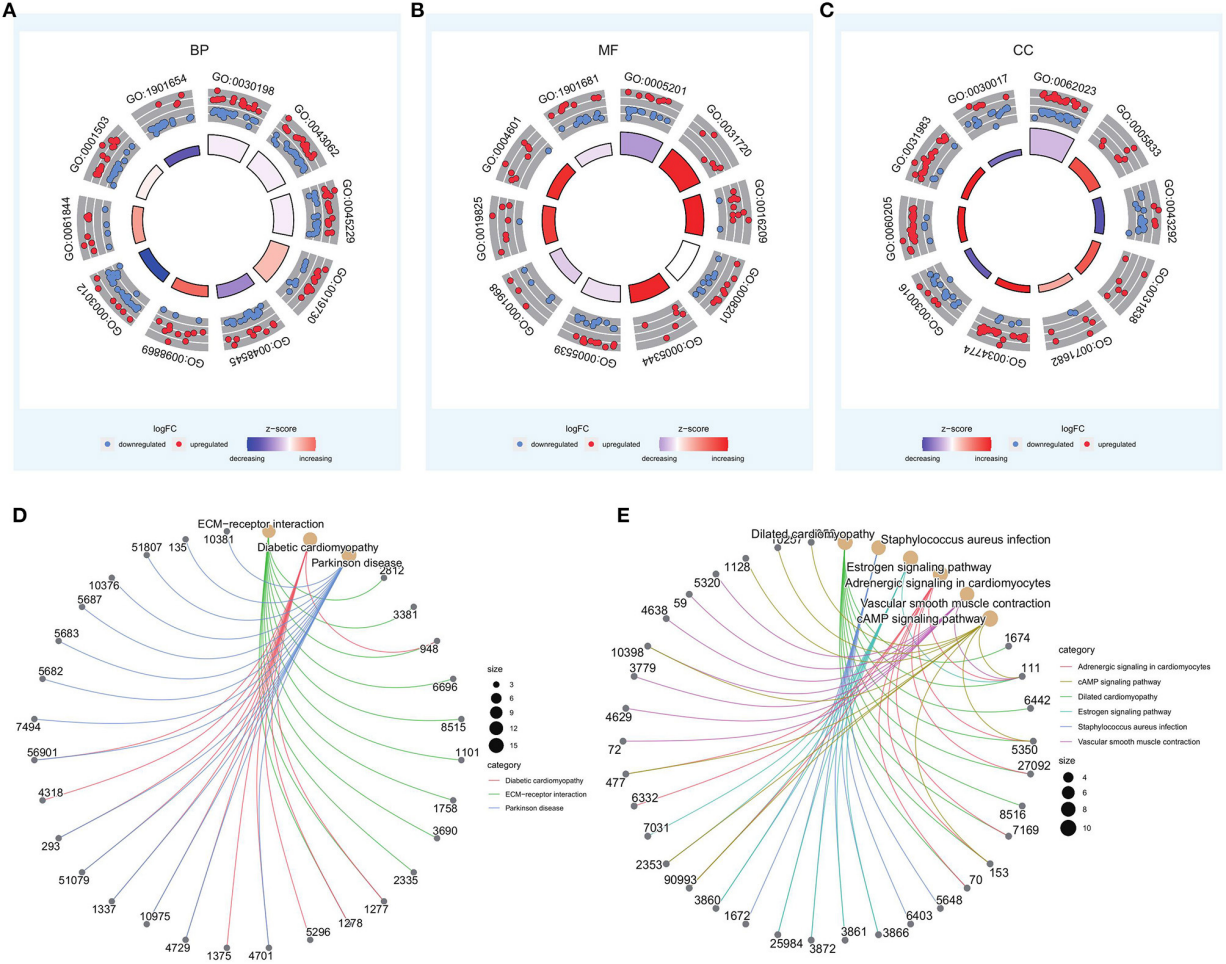
GO and KEGG enrichment analyses of differentially expressed genes in high- and low-risk groups. **(A–C)** Top 10 entries of biological process (BP), molecular function (MF), and cellular component (CC) in GO enrichment results. **(D,E)** Results of KEGG enrichment for upregulated and downregulated genes.

Of the identified 580 DEGs between the two groups, 314 were upregulated and 266 were downregulated. Subsequently, GO enrichment analysis and KEGG functional enrichment analysis were performed on the DEGs (Figure 5). Of the 187 GO entries that were enriched, BP accounted for 120 entries, MF for 33, and CC for 34 entries. BP was mainly enriched for extracellular organization, external encapsulating structure organization, antimicrobial humoral response, response to steroid hormone, and cellular oxidant detoxification. MF was mainly associated with extracellular matrix (ECM) structural constituent, haptoglobin binding, and antioxidant activity, whereas CC was associated with collagen-containing ECM, hemoglobin complex, and contaminant activity. For KEGG pathways, upregulated genes were enriched for ECM-receptor interaction, diabetic cardiomyopathy, and Parkinson's disease, whereas the downregulated genes were enriched for dilated cardiomyopathy, *Staphylococcus aureus* infection, estrogen signaling pathway, adrenergic signaling in cardiomyocytes, vascular smooth muscle contraction, and cAMP signaling pathway (Table 1).

**Table 1:** KEGG enrichment analysis.

**ID**	**Description**	**P.adjust**
**Enriched by upregulated DEGs**		
hsa04512	ECM-receptor interaction	0.000248
hsa05415	Diabetic cardiomyopathy	0.017731
hsa05012	Parkinson's disease	0.017731
**Enriched by downregulated DEGs**		
hsa05414	Dilated cardiomyopathy	0.001973
hsa05150	Staphylococcus aureus infection	0.007365
hsa04915	Estrogen signaling pathway	0.01166
hsa04261	Adrenergic signaling in cardiomyocytes	0.016359
hsa04270	Vascular smooth muscle contraction	0.027833
hsa04024	cAMP signaling pathway	0.043702

### GSEA

**Figure 6:**
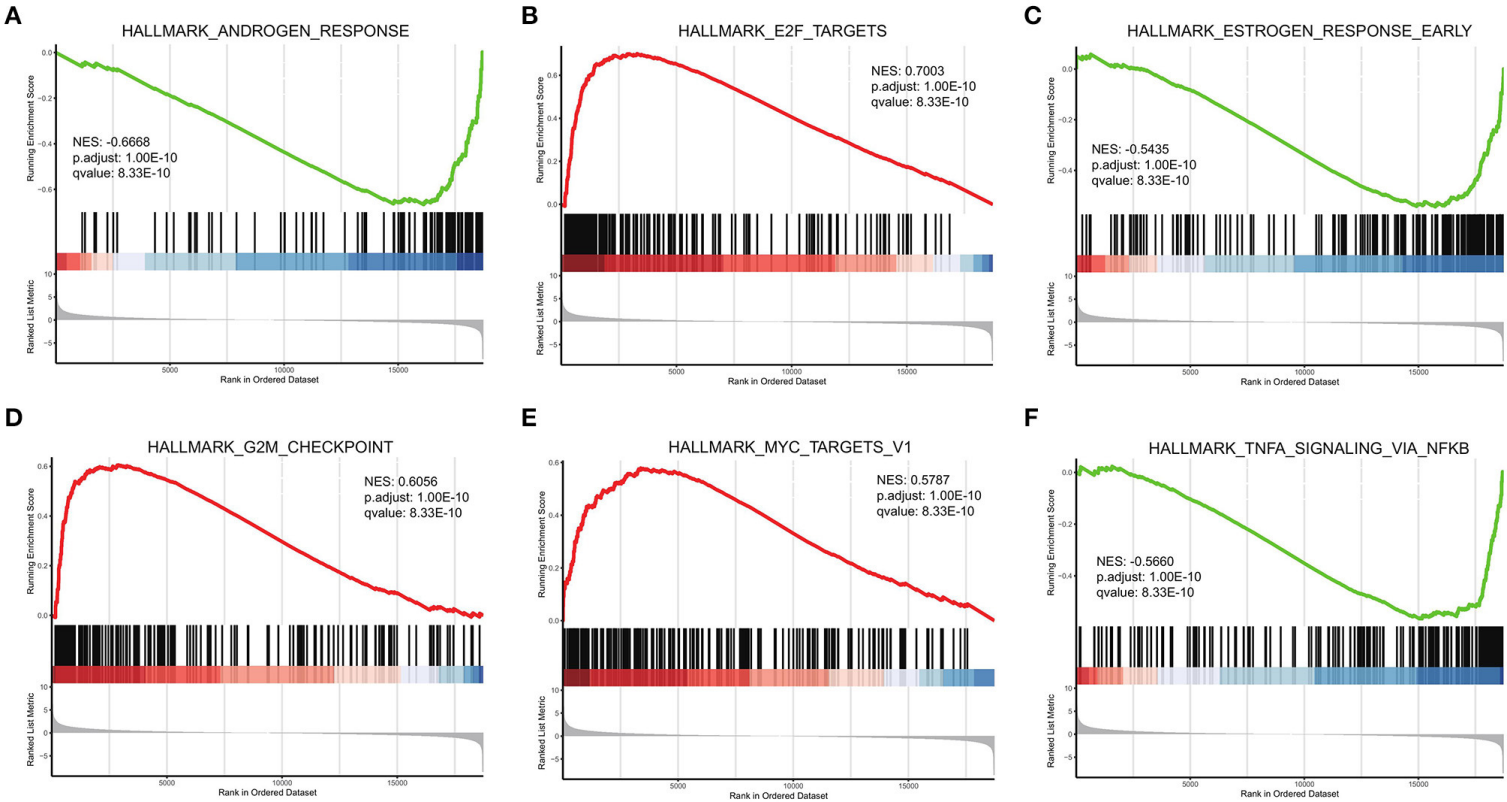
GSEA of high- and low-risk groups. **(A–F)** Top six results for high- and low-risk gene groups.

GSEA was performed based on the Hallmark gene set (Figure 6). The HALLMARK_ANDROGEN_RESPONSE was enriched to the low-risk group. In clinical practice, clinicians often use anti-androgen therapy to treat advanced prostate cancer where the disease has developed bone metastases (34). HALLMARK_MYC_TARGETS_V1 was enriched to the high-risk group. MYC overexpression has been reported to synergize with KRAS to induce aggressive hepatocellular carcinogenesis and metastasis (35).

**Figure 7:**
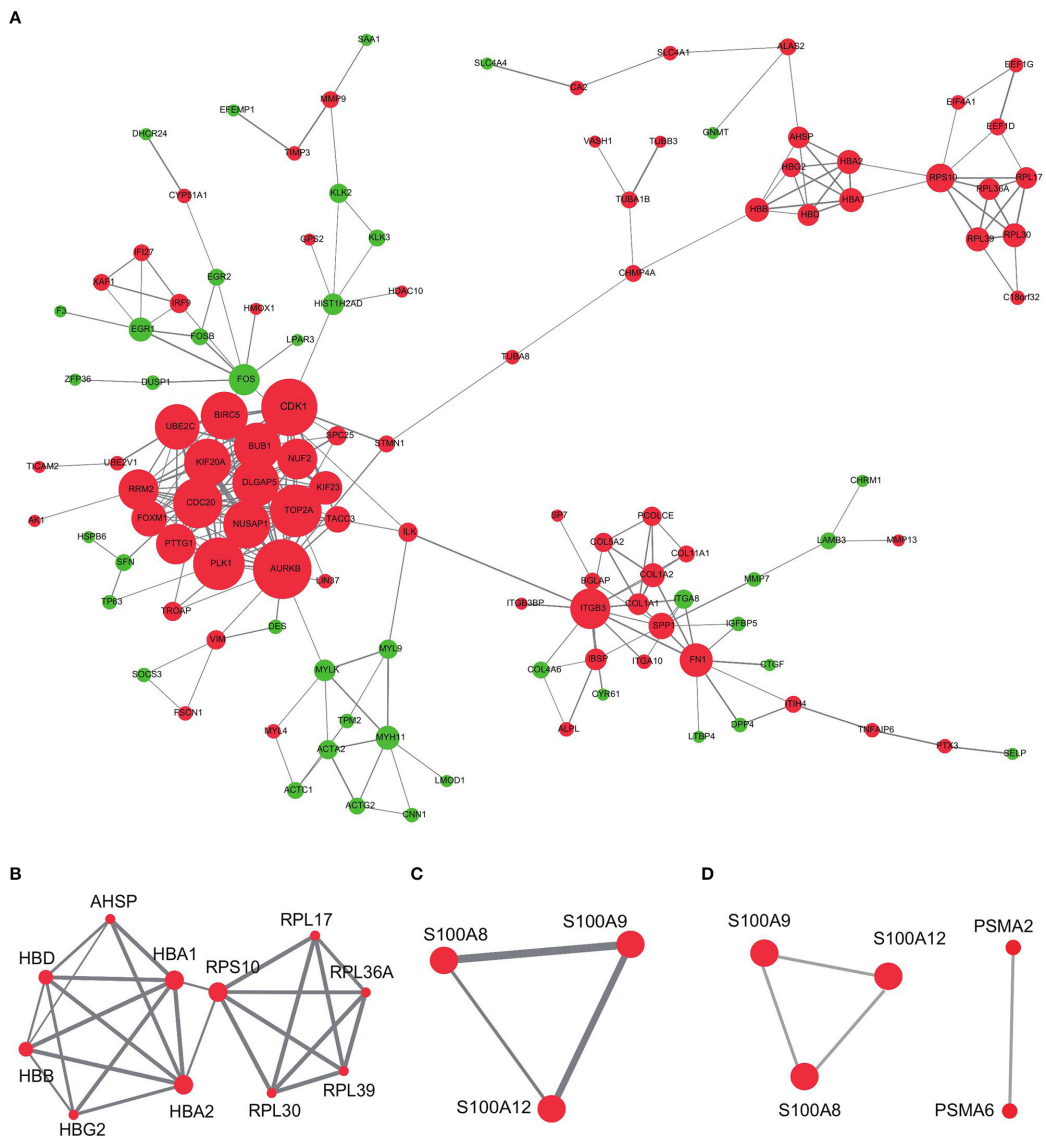
PPI network analysis of differentially expressed genes in high- and low-risk groups. **(A)** PPI networks of differential genes in high-risk and low-risk groups. **(B,C)** MCODE mining of modules 2 and 14. **(D)** Top five hub genes identified using the CytoHubba plugin. Red represents upregulation and green represents downregulation of expression. The size of the dots represents the degree in the network; the line thickness represents the combined interaction score between genes obtained from the STRING database.

We also investigated the internal interactions of bone metastasis-associated genes, mapping them to the PPI network using the STRING database. As a result (Figure 7A), the network PPI was enriched at *p* < 1.0e-16; we identified 355 pairs of reciprocal relationships corresponding to 202 network nodes. Mining of these data showed that module 2 (Figure 7B) contained the bone metastasis prognosis-related gene *RPL17*, which is overexpressed in breast cancer-associated brain metastases (36). Module 14 (Figure 7C) contained three genes: *S100A8, S100A9*, and *S100A12*. Among them, *S100A8* can promote bile duct cancer metastasis by upregulating VEGF expression through TLR4/NF-κB pathway activation (37). The hub node TOP5 mined by the CytoHubba plugin (Figure 7D) contained modules consisting of *S100A8, S100A9*, and *S100A12*, indicating the robustness of the association between *S100A8* expression and bone metastasis prognosis.

**Figure 8:**
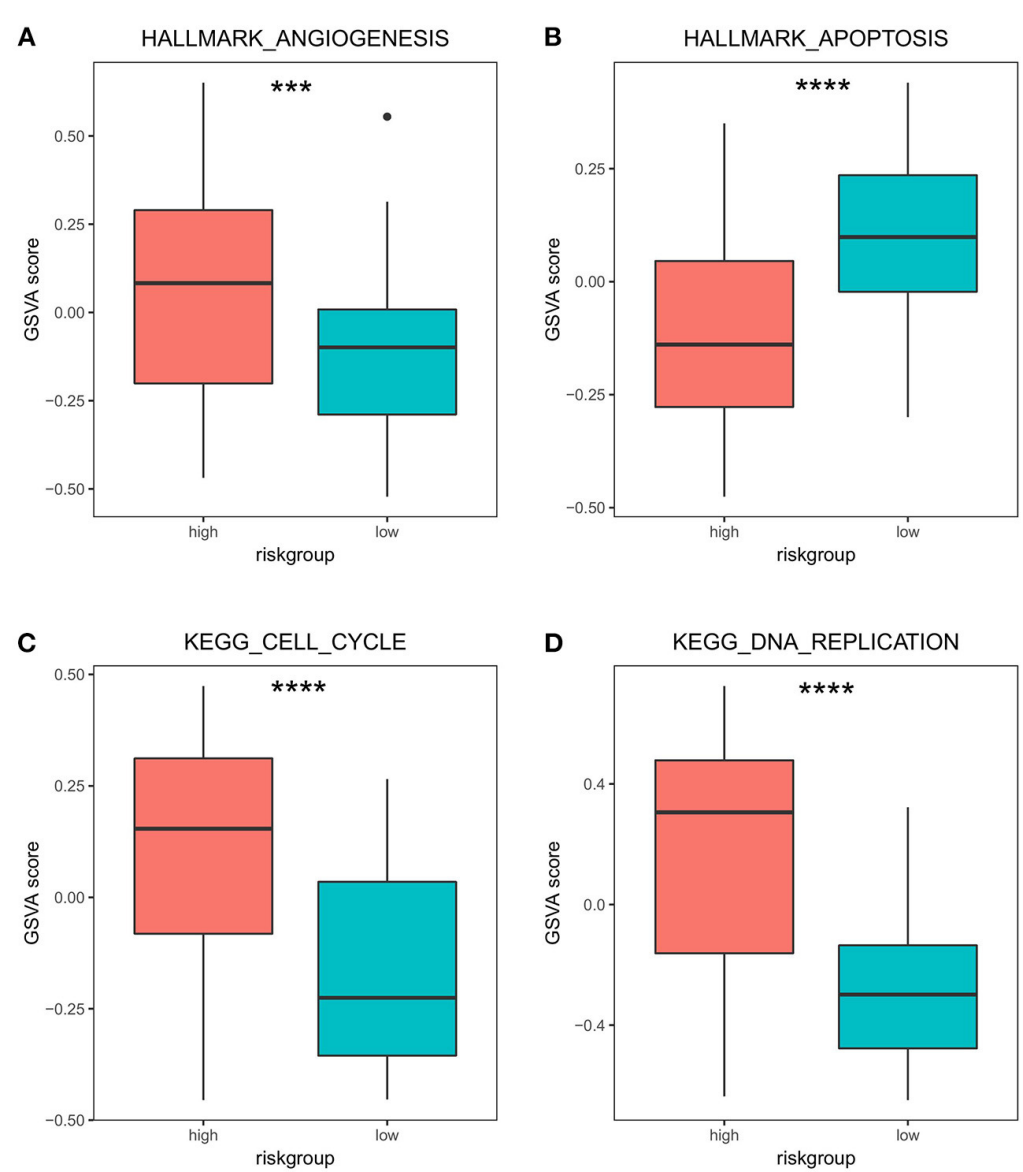
Differences in GSVA activity scores of transfer-related pathways in high- and low-risk groups. Differences in GSVA activity scores of cancer hallmarks: angiogenesis **(A)**, apoptosis **(B)**, cell cycle **(C)**, and DNA replication **(D)** in the high- and low-risk groups. *** *p* < 0.001; **** *p* < 0.0001.

In addition, we specifically focused on the pathways associated with PRAD bone metastasis (such as angiogenesis, apoptosis, cell cycle, DNA replication), and examined the differences in activity between high and low stratification groups in these pathways. The results (Figure 8) showed that the angiogenic activity was high in the high-risk group than in the low-risk group. Angiogenesis is necessary for aggressive tumor growth and metastasis and is an important link in controlling cancer progression (38); moreover, the high-risk group had lower apoptotic activity than the low-risk group. Escape from apoptosis is important for metastases (39, 40). Cell cycle and DNA replication activities were higher in the high-risk group than in the low-risk group. High gene replication rate is associated with the aggressiveness and metastasis of pancreatic cancer (41). Han et al. found that betulin could inhibit lung metastasis by inducing cell cycle arrest, autophagy, and apoptosis in metastatic colorectal cancer cells (42).

### Evaluation of the Prognostic Model

**Figure 9:**
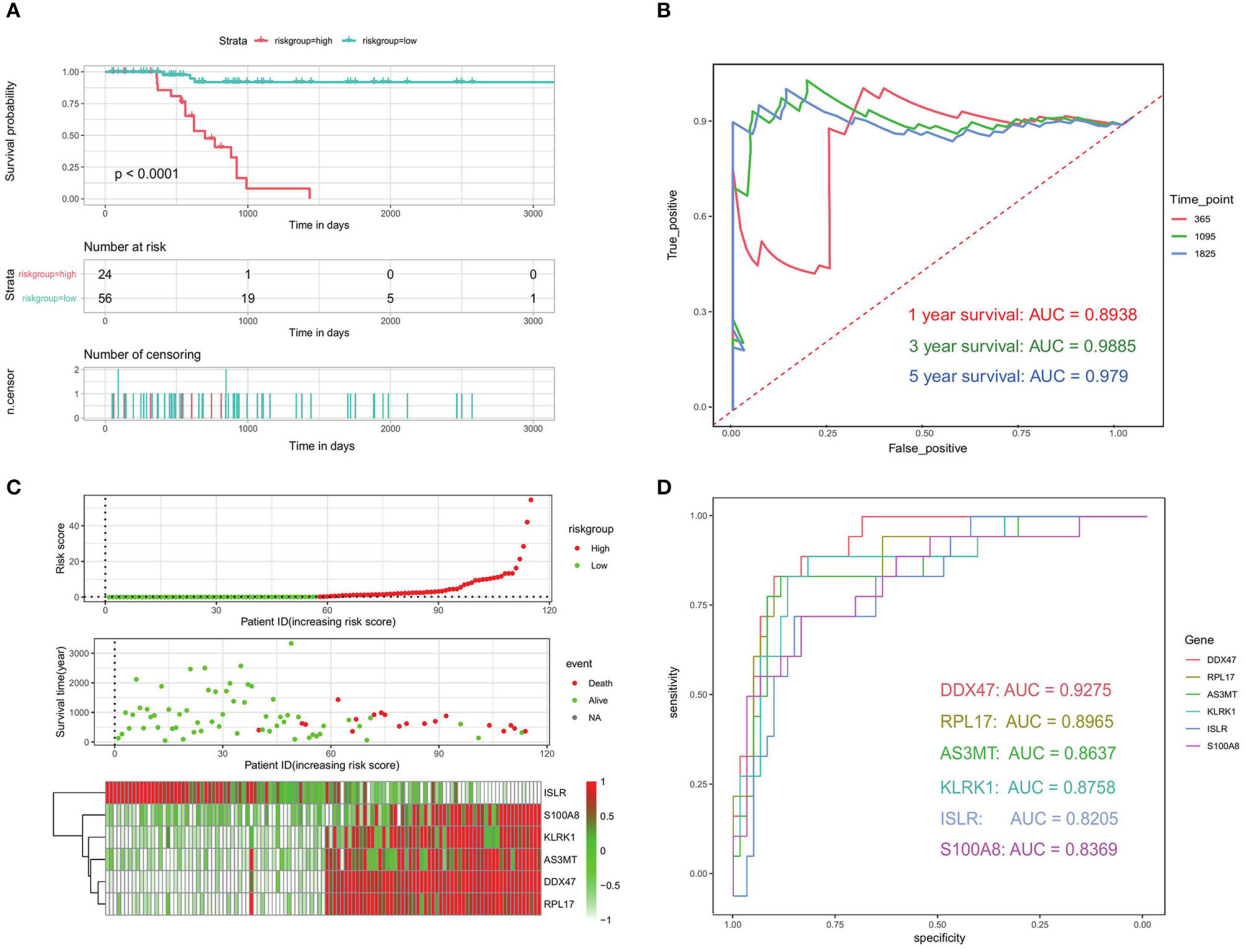
Evaluation of prognostic models related to bone metastases. **(A)** Kaplan-Meier analysis of patients in the high- and low-risk groups. **(B)** ROC analysis of risk scores to assess sensitivity and specificity. **(C)** Risk factor analysis to assess the relationship among risk score, death, and characteristic gene expression. **(D)** ROC analysis of the six identified bone metastasis-associated prognostic genes.

We used a survival difference analysis to compare the high- and low-risk groups and assess the effectiveness of the prognostic model. The results revealed that patients in the high-risk group had a considerably worse outcome than those in the low-risk group (*p* < 0.0001; Figure 9A). Specifically, the median OS for patients in the high-risk group was 622 days, compared to 844 days for patients in the low-risk group. To further estimate the predictive performance of this risk model, time-dependent ROC analyses were performed for 1-year, 3-year, and 5-year OS. They corresponded to the AUC values of 0.8938, 0.9885, and 0.979, respectively, which demonstrated the good performance of our model (AUC > 0.5; Figure 9B). Except for *ISLR*, the higher the risk score, the higher the expression of the genes associated with it, and the earlier the patient death event occurred (Figure 9C). To compare the reliability of our prognostic model consisting of six genes, we compared the ROC analysis of individual genes. The results showed (Figure 9D) that the six-gene model had better prognostic prediction ability than the single-gene model. These results suggest that the established prognostic model is valid and related to bone metastasis in PRAD.

### Validation of the Prognostic Model

**Figure 10:**
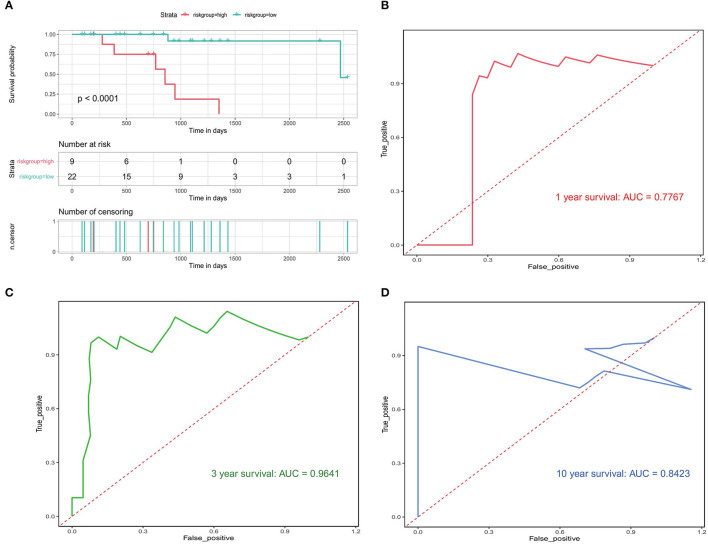
Validation of bone metastasis-associated prognostic models. **(A)** Kaplan-Meier analysis of the high first risk group in the validation set. **(B–D)** Validation of time-dependent ROC analyses focused on 1, 3, and 10 years.

We also used the validation set to check the accuracy of our prognostic model. A significant difference in median OS between the high- and low-risk groups (750 vs. 910 days; *p* < 0.0001) was observed (Figure 10A), which was in line with the results from the training set. The results of the validation data showed that the AUC for 1, 3, and 10-year OS was 0.7767, 0.9641, and 0.8423, respectively (Figures 10B–D). These results indicate that our PRAD bone metastasis-related risk model is robust.

### Prognostic Independence of the Validation Model

**Figure 11:**
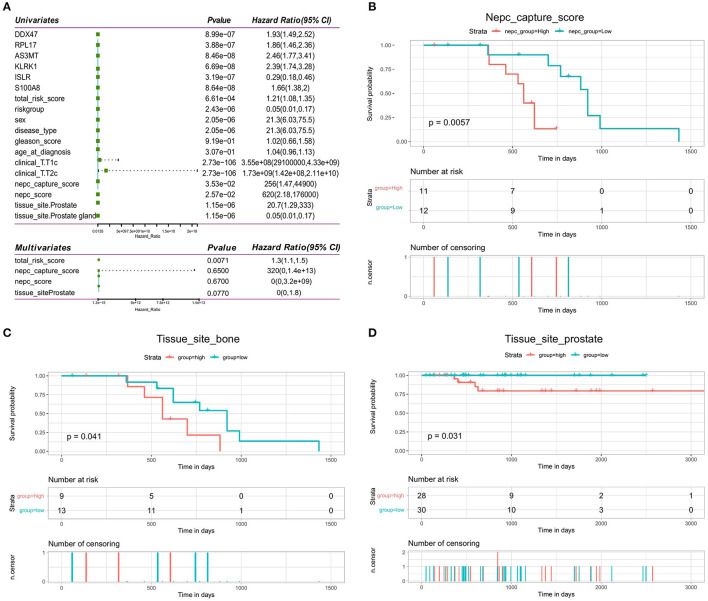
Validation of independence of bone metastasis-related prognostic models. **(A)** Forest plot of single-factor regression analysis and multifactor regression analysis. **(B)** Kaplan-Meier analysis of prognosis for the high- and low-NEPC score groups. **(C,D)** Kaplan-Meier analysis of the high- and low-risk groups for the clinical factor tissue site (bone and prostate).

We investigated whether the clinical characteristics and risk scores of PRAD patients in the training set were associated with prognosis. The results of univariate Cox regression analysis showed that risk score, T-stage, NEPC score, tissue site, and OS were significantly associated. These four factors were used as covariates in a multivariate Cox regression analysis, which showed that risk score (HR = 1.3, 95% CI = 1.1–1.6, *p* < 0.05) was an independent prognostic factor for OS in patients (Figure 11A). The results of univariate and multifactorial Cox analyses showed that NEPC score and tissue site were risk factors for the prognosis, although these were not independent risk factors. Then, we performed a prognostic analysis of NEPC score (continuous variable) and tissue site (discrete variable). We divided the data into two groups according to the median value and found that the group with higher NEPC score had a lower survival rate (Figure 11B). For tissue site, we looked at the prognosis in the high- and low-risk groups individually for each classification. The results (Figures 11C,D) showed that the risk score is an independent prognostic factor regardless of the tissue site, which further illustrates the independent prognostic value of the risk model.

### Construction and Evaluation of Column Line Graphs

**Figure 12:**
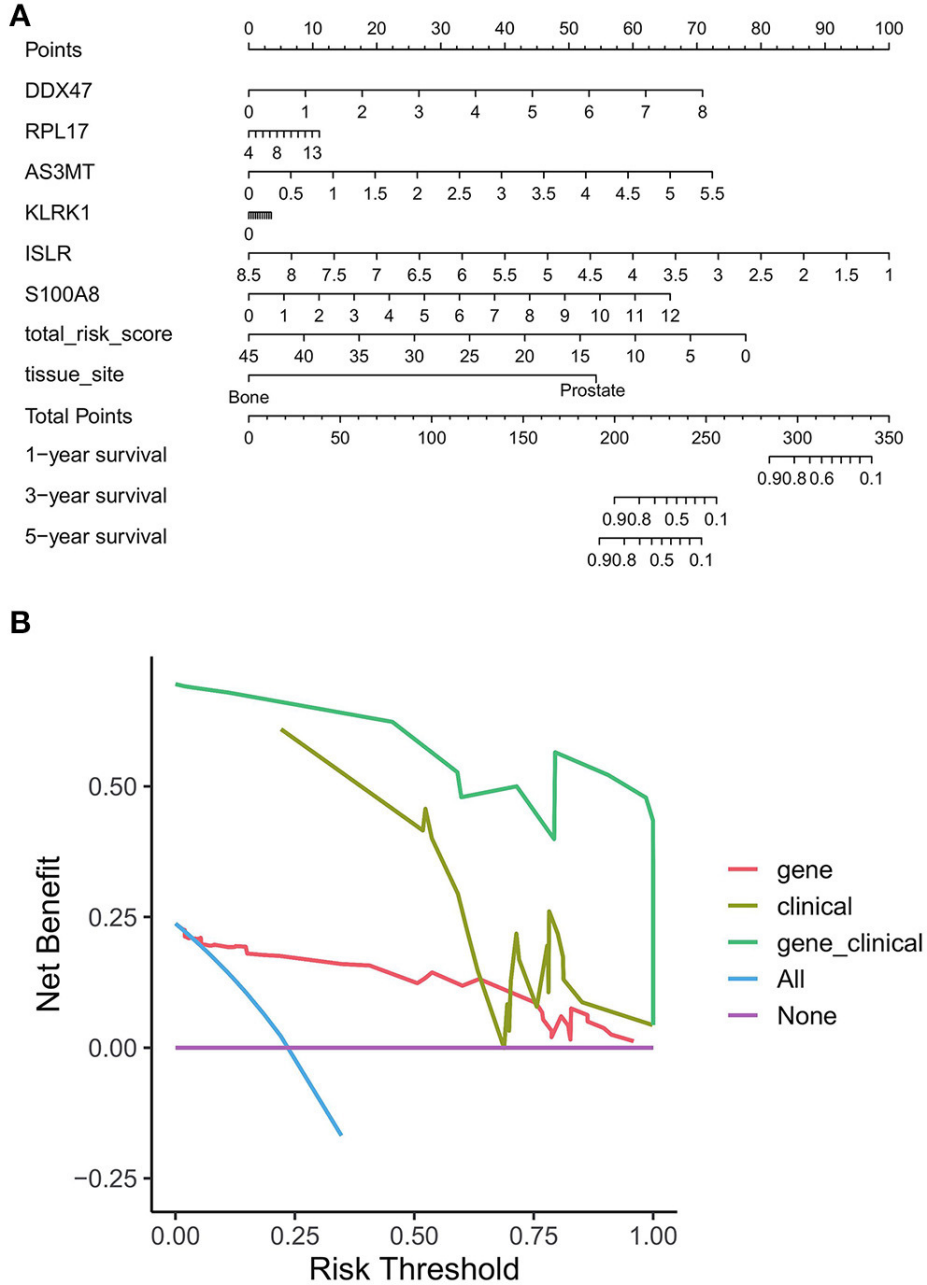
Benefit assessment of bone metastasis-related prognostic models applied to the clinical setting. **(A)** Nomogram with bone metastasis prognostic genes and clinical factors for predicting OS in PRAD. **(B)** DCA of benefit rates for models of bone metastasis prognostic genes and clinical factors vs. clinical factor models, and bone metastasis prognostic genes.

To evaluate whether our model can effectively predict the prognosis of PRAD in a clinical setting, we incorporated factors associated with the OS of PRAD (risk score combined with clinicopathological characteristics) into the model and constructed a column line plot (Figure 12A) to predict the 1, 3, and 5-year OS patients. The column line plot model validated the reliability of the model and has prospective clinical applications. On performing DCA of PRAD patients —to assess the utility of the model in clinical application after incorporating clinical factors—we found that the inclusion of clinical factors significantly increased patient benefits (Figure 12B).

### Immunohistochemistry(IHC) of Proteins Encoded by Bone Metastasis-Related Prognostic Genes

**Figure 13:**
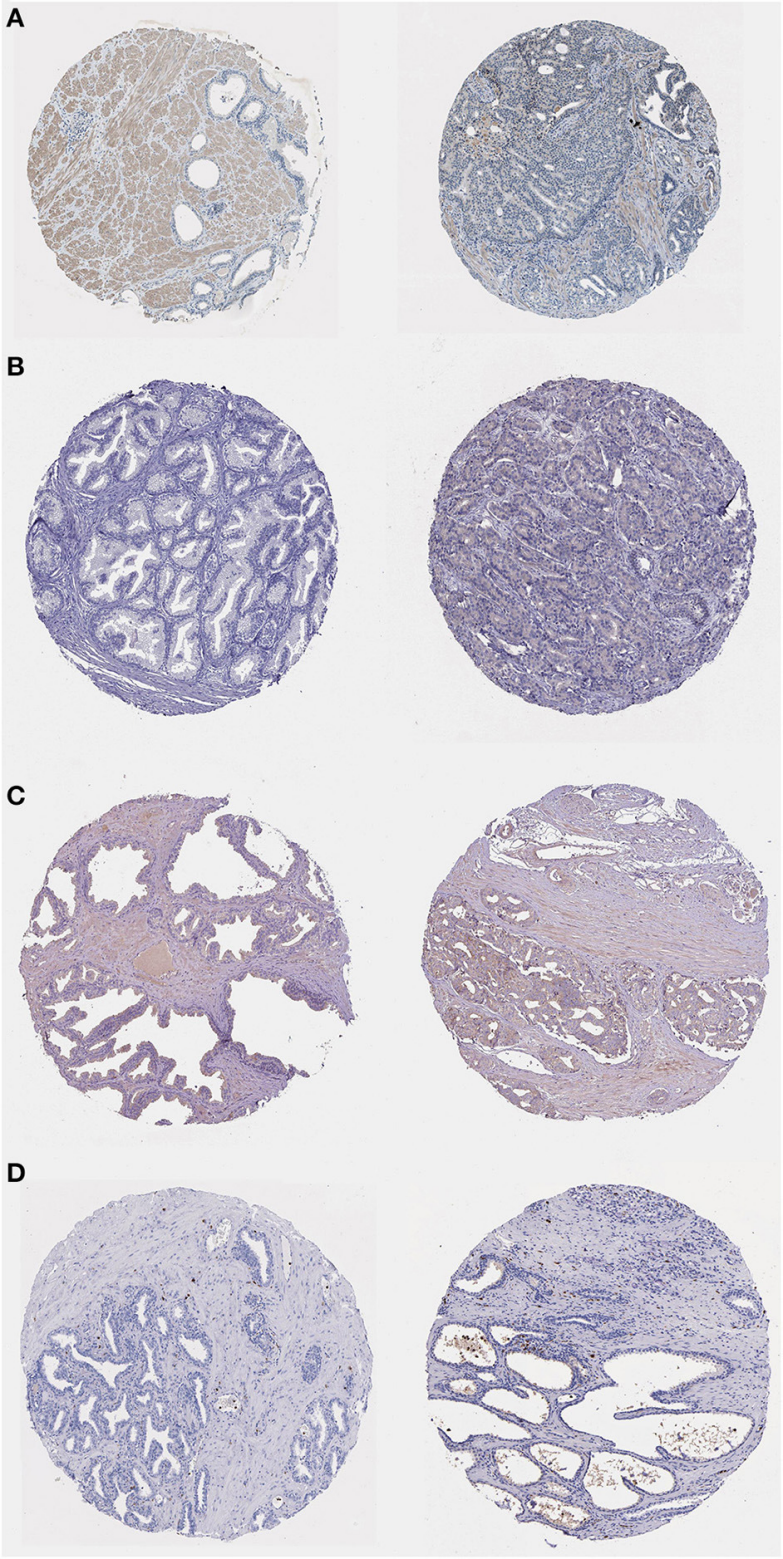
Immunohistochemistry of bone metastasis-associated prognostic genes in normal prostate tissue (left) and PRAD (right) samples. **(A)** Immunohistochemistry of DDX47 in normal prostate tissue (left) and PRAD (right). Patient information: normal (patient id: 2,053, male, age 51); PRAD (patient id: 3,571, male, age 71). **(B)** Immunohistochemistry of AS3MT in normal prostate tissue (left) and PRAD (right) samples. Patient information: normal (patient id: 3,376, male, age 72); PRAD (patient id: 5,412, male, age 63). **(C)** Immunohistochemistry of ISLR in normal prostate tissue (left) and PRAD (right) samples. Patient information: normal (patient id: 3,497, male, age 37); PRAD (patient id: 4,365, male, age 71). **(D)** Immunohistochemistry of S100A8 in normal prostate tissue (left) and PRAD (right). Patient information: normal (patient id: 2,053, male, age 51); PRAD (patient id: 3,190, male, age 72) samples.

To determine the expression of bone metastasis-prognostic genes in PRAD, we searched the Human Protein Atlas database (https://www.proteinatlas.org/) (43) for immunofluorescence data of the proteins encoded by the six identified genes (Figure 13). DDX47 expression was not detected in the normal prostate tissue, Medium intensity staining was detected in PRAD (Figure 13A). The results indicated low expression of AS3MT in PRAD tissue, but not detected in the normal tissue (Figure 13B); HPA050811 antibody was used for ISLR (Figure 13C), in PRAD, expression of ISLR was moderate the normal prostate tissue was low; HPA002791 antibody was used for analyzing the expression of S100A8, the expression of S100A8 was not detected in the normal prostate tissue, however, S100A8 expression was detected in PRAD (Figure 13D). The results of immunohistochemical analysis of these genes in the HPA database (Table 2).

**Table 2:** IHC validation in HPA.

**Gene**	**Antibody**	**Tissue**	**Staining**	**Intensity**	**Quality**	**Location**
DDX47	HPA014855	Nomal	Not detected	Weak	<25%	Nuclear
		PRAD	Medium	Strong	<25%	Nuclear
AS3MT	HPA017856	Nomal	Not detected	Negative	None	None
		PRAD	Low	Weak	75%-25%	Cytoplasmic/membranous
SLR	HPA050811	Nomal	Low	Weak	75%-25%	Cytoplasmic/membranous
		PRAD	Medium	Moderate	>75%	Cytoplasmic/membranous
S100A8	HPA002791	Nomal	Not detected	Negative	None	None
		PRAD	Low	Moderate	<25%	Cytoplasmic/membranous

## Discussion

Approximately 64% of patients with advanced prostate cancer have bone metastases (44), which are linked to increased risks of morbidity and mortality (45), and poor prognosis (46). Many researchers are trying to establish a clinical trial protocol/methodto map disease progression. However, owing to the lack of clinical indicators that accurately represent disease progression, it is difficult to predict the prognosis of patients with PRAD bone metastases. Individual differences have a significant impact on treatment success in PRAD patients with bone metastases. The Discovery of new biomarkers and prognostic indicators to understand the mechanisms involved in the development of PRAD with bone metastases is therefore critical for proper disease diagnosis and therapy. We analyzed 82 primary samples and 82 bone metastasis PRAD samples in this study. mRNA expression was determined using genome-wide microarray data, yielding a total of 563 PRAD bone metastasis-related genes. Of these, 375 OS-related bone metastasis-related genes were obtained using univariate Cox regression analysis, and these potential prognostic genes were further evaluated using Lasso Cox regression analysis. A six-gene model performed better than a single-gene model in terms of prognostic power. Univariate Cox regression analysis showed that the risk score, T-stage, NEPC score, tissue site, and OS were all significantly linked. GO and KEGG enrichment showed that the DEGs were largely related to collagen-containing ECM, hemoglobin complex binding bead protein, and antioxidant activity contractile fibrils.

In the first phase of this study, to investigate the biological differences between the high- and low-risk groups, 580 DEGs were identified; of these, 314 were upregulated genes and 266 were downregulated genes. GO and KEGG enrichment analyses were then performed to identify the BP associated with DEGs involved in PRAD bone metastases. Among the BP annotations, ECM tissue, extracellular structural tissue, outer envelope structural tissue, antimicrobial humoral response, steroid hormone response, and cellular oxidative detoxification were all significantly linked to the development of PRAD bone metastasis. Prostate cancer cells, osteoblasts, osteoclasts, bone stroma, and endothelial cells interact in complicated ways during bone metastasis (47). In an intraosseous prostate cancer xenograft model, overexpression of estrogen related receptors was reported to increase prostate cancer cell proliferation in bones by activating osteoblasts and controlling ECM protein secretion in the stroma (48). Matrix metalloproteinase-9 is involved in prostate cancer metastasis via its role in epithelial to mesenchymal transition and the degradation of the ECM. Only the p53-like isoform, which is enriched in ECM biomarkers and cancer-associated fibroblasts, has been demonstrated to cause bone metastases (49). The prostate is an organ that responds to sex steroid hormones (50), including androgens, which affect the development of male sexual traits and reproductive function, are important regulators of prostate cancer cell growth and proliferation (51, 52), and are responsible for the clinical progression of prostate cancer (53). Prostate cancer research has shown that steroid hormones stimulate cancer cell proliferation and invasiveness (54). Endogenous steroid hormones and the cellular response to hormones have been identified as cancer prevention targets, indicating that endogenous risk factors can be modified (51).

In the second phase of this study, the biological function of DEGs associated with PRAD bone metastases was investigated using GSEA. Upregulated genes were enriched for ECM-receptor interactions, diabetic cardiomyopathy, Parkinson's disease, and downregulated genes were enriched for dilated cardiomyopathy, *Staphylococcus aureus* infection, estrogen signaling pathway, adrenergic signaling in cardiomyocytes, vascular smooth muscle contraction, and cAMP signaling pathway. The 'ECM-receptor interaction' pathway is known to contribute significantly to the development and spread of prostate cancer (55) integrin alpha V beta 3 is abundantly expressed in the vascular matrix and is upregulated in advanced stages of prostate cancer (56). Moreover, it is thought to be necessary for prostate tumor invasion and bone metastases (57). Development of prostate cancer results in the alteration of ECM composition and the cellular receptors for ECM ligands (58). The cAMP signaling pathway is an important signaling pathway in biological systems (59), and abnormal cAMP signaling is closely linked to prostate cancer progression (60). In an *in vitro* study, researchers used a specific isoform of the DN-PDE4 beta-inhibitory protein to alter the perinuclear cAMP signaling, resulting in desensitization of the β2-adrenergic receptor and increased cell growth (61).

The PPI network identified in this study showed multiple interactions, with 355 pairs of reciprocal relationships corresponding to 202 network nodes. Further detailed study of these genes may reveal the pathophysiological mechanisms of PRAD in bone metastases. The pro-apoptotic protein calmodulin A/B (S100A8/9) is produced by immune cells and can also be released by post-hypoxic necrosis of tumor cells (in actively growing tumors). Interestingly, *S100A8/A9* expression has also been linked to tumor development, invasion, or metastasis (62, 63). Tumor cell migration and invasion are facilitated by the upregulation of matrix metalloproteinase expression and inhibited by the downregulation of *S100A8* or *S100A9*, which correlates with their abnormal expression in many cancer types (64–66). Targeting *S100A8* and *S100A9* may help stop tumor cells from migrating to places where they can spread (67). Upregulation of *S100A8/A9* occurs as a result of immune cells or the tumor itself infiltrating the tumor microenvironment, helping to create a pre-metastatic milieu (68). For example, increased expression of *S100A8* in prostate cancer models was found to alter the tumor stroma (69).

Six prognostic genes, *DDX47, PRL17, AS3MT, KLRK1, ISLR*, and *S100A8*, were discovered to have prognostic value in the study. The survival disparities between the high- and low-expression groups for each prognostic gene were compared. Five prognostic genes *DDX47, RPL17, AS3MT, KLRK1*, and *S100A8* were found to be unfavorable for the survival of PRAD bone metastases; their expression was considerably higher in the high-risk group than in the low-risk group. The *ISLR* gene was found to be a favorable factor for PRAD bone metastasis survival, with the survival rate in the high-risk group being much lower than that in the low-risk group. Time-dependent ROC studies performed for the 1-, 3-, and 5-year OS for quantifying the predictive performance of this risk model resulted in AUC values of 0.8938, 0.9885, and 0.979, respectively, showing that our model performed well. We performed ROC analyses of individual genes to compare the reliability of the six-gene prognostic model. The results revealed that the six-gene model outperformed the single-gene model in terms of prognostic power. All these findings point to the development of a reliable prognostic model for PRAD bone metastases.

Our study has certain limitations. First, while microarray-based bioinformatics analysis is a powerful tool for understanding molecular mechanisms and identifying potential biomarkers of PRAD bone metastasis, more experimental studies, such as those using real-time PCR, western blot, immunohistochemical analysis, and cellular and animal experiments, are needed to elucidate the role of key genes and the underlying mechanisms of PRAD bone metastasis. Second, functional investigations on the activities of DEGs and hub genes in PRAD bone metastases in terms of tissue-type specificity and cell-type specificity are still needed. PRAD bone metastasis signaling networks are more complex than previously assumed, including estrogen signaling pathways, vascular smooth muscle contraction, and signaling pathways. Investigation of molecular mechanisms is also needed to provide more precise and robust evidence for the putative genes and pathways related to the PRAD bone metastasis prediction genes. Finally, this study is limited to single histology, which does not provide a complete picture of gene function. Therefore, multi-omics studies, particularly at the protein and functional levels, are needed to fully understand the relevance of the identified genes.

In conclusion, we aimed to investigate the molecular mechanisms driving PRAD bone metastasis progression using a complete bioinformatics analysis to discover the key biological functions and pathways involved. We performed PPI network analysis, functional similarity analysis, ROC curve analysis, Kaplan-Meier survival analysis, and Cox regression analysis for exploring six additional candidate genes for use as diagnostic biomarkers. The findings of the present study need further validation through molecular biological studies.

## Data Availability Statement

The original contributions presented in the study are included in the article/Supplementary Material, further inquiries can be directed to the corresponding author/s.

## Ethics Statement

The patient information designed in this study can be obtained through an online database and does not require an ethical statement.

## Author Contributions

WL, XH, and JC: data curation. WL and YD: formal analysis. KH and JQ: methodology. XH: writing—original draft. JC and WL: writing—review and editing. All authors contributed to the article and approved the submitted version.

## Conflict of Interest

The authors declare that the research was conducted in the absence of any commercial or financial relationships that could be construed as a potential conflict of interest.

## Publisher's Note

All claims expressed in this article are solely those of the authors and do not necessarily represent those of their affiliated organizations, or those of the publisher, the editors and the reviewers. Any product that may be evaluated in this article, or claim that may be made by its manufacturer, is not guaranteed or endorsed by the publisher.

## Abbreviations

BP: Biological Process
MF: molecular function
CC: molecular function
TCGA: The Cancer Genome Atlas
GO: Gene Ontology
KEGG: Kyoto Encyclopedia of Genes and Genomes.
