# Supplementary material for: A Novel Overall Survival Prediction Signature Based on Comprehensive Research in Prostate Cancer Bone Metastases
Source: Front Med (Lausanne). 2022 Jun 16;9:815541. doi: 10.3389/fmed.2022.815541 (PMC9243502; doi:10.3389/fmed.2022.815541)
Supplement: Supplementary file 1 [file Table_1.doc]

**Supplementary table**

**Differentially expressed genes related to bone metastases of prostate cancer**

| Gene | logFC | P.Value | adj.P.Val |
| --- | --- | --- | --- |
| MATR3 | 5.657278 | 1.44E-55 | 2.70E-51 |
| CNN1 | -6.4012 | 3.37E-55 | 3.16E-51 |
| ACTG2 | -7.381 | 1.71E-54 | 1.07E-50 |
| XBP1 | 8.318877 | 2.19E-53 | 1.03E-49 |
| LUC7L2 | 6.246205 | 9.16E-53 | 3.43E-49 |
| TCF21 | -3.30724 | 3.48E-52 | 9.54E-49 |
| EEF1G | 9.570829 | 3.57E-52 | 9.54E-49 |
| DES | -8.76793 | 1.80E-51 | 4.22E-48 |
| ABHD16A | 2.822742 | 7.53E-51 | 1.57E-47 |
| TOMM6 | 8.218967 | 1.11E-50 | 1.90E-47 |
| ZC3H11A | -5.26155 | 1.12E-50 | 1.90E-47 |
| PIK3R2 | 4.731827 | 1.64E-50 | 2.56E-47 |
| PCP4 | -5.4367 | 2.51E-50 | 3.62E-47 |
| SERINC4 | 4.237194 | 3.39E-50 | 4.53E-47 |
| IFI30 | 6.717327 | 4.55E-50 | 5.66E-47 |
| FOXF1 | -3.89284 | 4.83E-50 | 5.66E-47 |
| NME2 | 6.839384 | 3.09E-49 | 3.40E-46 |
| GP1BB | 4.094698 | 3.64E-49 | 3.79E-46 |
| TOP3B | 3.428732 | 1.27E-48 | 1.25E-45 |
| MFAP4 | -4.84159 | 1.35E-48 | 1.27E-45 |
| MOXD1 | -3.85656 | 2.14E-48 | 1.91E-45 |
| ANKHD1 | 3.915904 | 2.36E-48 | 2.01E-45 |
| TEN1 | 4.829517 | 2.88E-48 | 2.35E-45 |
| CD68 | 5.997888 | 3.37E-48 | 2.63E-45 |
| ATRIP | 3.304175 | 4.44E-48 | 3.31E-45 |
| EIF4A1 | 6.498253 | 4.59E-48 | 3.31E-45 |
| MYH11 | -5.71019 | 5.29E-48 | 3.67E-45 |
| RTEL1 | 3.176868 | 7.51E-48 | 5.03E-45 |
| DDX47 | 4.571756 | 8.99E-48 | 5.81E-45 |
| IBSP | 6.88494 | 2.32E-47 | 1.45E-44 |
| ARL6IP4 | 5.09411 | 2.82E-47 | 1.70E-44 |
| C17orf49 | 4.4682 | 5.70E-47 | 3.34E-44 |
| SPNS1 | 4.135915 | 5.89E-47 | 3.35E-44 |
| PTRH1 | 3.153242 | 1.12E-46 | 6.15E-44 |
| SARNP | 4.842521 | 1.85E-46 | 9.90E-44 |
| PSMA2 | 4.73649 | 2.69E-46 | 1.40E-43 |
| GET4 | 3.346439 | 4.26E-46 | 2.15E-43 |
| SCLY | 2.583154 | 5.25E-46 | 2.59E-43 |
| RBM34 | 3.74462 | 9.42E-46 | 4.53E-43 |
| ZNF410 | 3.642894 | 1.94E-45 | 9.08E-43 |
| RNASEK | 4.017337 | 2.22E-45 | 1.01E-42 |
| HYI | -3.76719 | 2.74E-45 | 1.22E-42 |
| PSMA6 | 3.880664 | 6.82E-45 | 2.97E-42 |
| ZNF564 | 2.663635 | 1.10E-44 | 4.68E-42 |
| NDUFA7 | 4.698859 | 1.46E-44 | 6.08E-42 |
| ABHD16B | 3.297742 | 1.87E-44 | 7.61E-42 |
| LMOD1 | -4.59856 | 3.03E-44 | 1.21E-41 |
| PSMA1 | 3.051063 | 3.10E-44 | 1.21E-41 |
| TMEM110 | 2.760863 | 4.06E-44 | 1.55E-41 |
| TREX1 | 4.493921 | 6.72E-44 | 2.52E-41 |
| SYNPO2 | -4.55706 | 8.21E-44 | 3.02E-41 |
| ZNF177 | 2.978938 | 1.10E-43 | 3.95E-41 |
| SNX15 | 3.80735 | 1.29E-43 | 4.57E-41 |
| LTC4S | 4.107647 | 1.47E-43 | 5.09E-41 |
| ZNF625 | 2.114002 | 3.78E-43 | 1.29E-40 |
| CMC4 | 3.405886 | 4.25E-43 | 1.42E-40 |
| FLNC | -4.65997 | 5.16E-43 | 1.70E-40 |
| POLR2F | 4.299181 | 5.96E-43 | 1.92E-40 |
| GTF2H4 | 3.050237 | 7.08E-43 | 2.25E-40 |
| TUBA1B | 3.039919 | 8.14E-43 | 2.54E-40 |
| KRTCAP2 | 4.26499 | 8.29E-43 | 2.54E-40 |
| SPSB3 | 4.352833 | 8.98E-43 | 2.71E-40 |
| CRIP1 | 5.01402 | 1.01E-42 | 3.00E-40 |
| C7orf25 | 2.841179 | 1.61E-42 | 4.70E-40 |
| BSCL2 | 2.700303 | 4.51E-42 | 1.30E-39 |
| TXNDC5 | 4.413042 | 4.66E-42 | 1.32E-39 |
| ELP4 | -2.55682 | 4.78E-42 | 1.32E-39 |
| TTC4 | 2.706565 | 4.78E-42 | 1.32E-39 |
| PGAM2 | 4.16207 | 5.82E-42 | 1.58E-39 |
| TARDBP | 2.374841 | 1.07E-41 | 2.87E-39 |
| DNLZ | 2.453216 | 1.15E-41 | 3.02E-39 |
| PAGR1 | 3.523439 | 1.16E-41 | 3.02E-39 |
| LRCH4 | 2.650989 | 1.30E-41 | 3.33E-39 |
| PMM2 | 2.619298 | 1.42E-41 | 3.60E-39 |
| AARSD1 | 3.006917 | 2.11E-41 | 5.26E-39 |
| CYP51A1 | 3.517773 | 2.16E-41 | 5.33E-39 |
| COX20 | 3.113441 | 2.60E-41 | 6.33E-39 |
| TOMM5 | 2.827304 | 2.65E-41 | 6.36E-39 |
| CTAGE5 | 2.431012 | 7.35E-41 | 1.74E-38 |
| NPFF | 3.195453 | 8.44E-41 | 1.98E-38 |
| C11orf52 | 3.674742 | 9.34E-41 | 2.16E-38 |
| MRPL53 | 3.034238 | 1.19E-40 | 2.71E-38 |
| PGM5 | -3.85262 | 1.28E-40 | 2.89E-38 |
| ICAM3 | 3.923501 | 1.50E-40 | 3.34E-38 |
| LRRN4CL | -2.34889 | 7.68E-40 | 1.65E-37 |
| ADIRF | 6.357926 | 1.51E-39 | 3.15E-37 |
| HBB | 7.868533 | 1.59E-39 | 3.27E-37 |
| MRGPRF | -3.53061 | 1.61E-39 | 3.27E-37 |
| CNTN1 | -2.98225 | 2.04E-39 | 4.10E-37 |
| PCDH10 | -2.74082 | 2.16E-39 | 4.30E-37 |
| PILRB | 3.98954 | 2.41E-39 | 4.76E-37 |
| UBE2V1 | 3.666335 | 2.81E-39 | 5.48E-37 |
| WDR73 | 2.471515 | 3.05E-39 | 5.89E-37 |
| JMJD7 | 3.913052 | 3.79E-39 | 7.24E-37 |
| H2AFY | 2.180173 | 4.06E-39 | 7.68E-37 |
| ITIH4 | 2.579926 | 4.15E-39 | 7.77E-37 |
| NDST2 | 2.88402 | 4.52E-39 | 8.38E-37 |
| WFDC2 | -4.76735 | 4.71E-39 | 8.65E-37 |
| CNPY2 | 2.076637 | 5.00E-39 | 9.10E-37 |
| SLC25A10 | 2.842169 | 5.57E-39 | 1.00E-36 |
| ZNF23 | 2.111601 | 6.84E-39 | 1.21E-36 |
| MRPL38 | 3.137246 | 7.59E-39 | 1.33E-36 |
| GPS2 | 3.56856 | 8.33E-39 | 1.44E-36 |
| RPL39 | 4.437891 | 8.39E-39 | 1.44E-36 |
| RPL41 | 2.794512 | 1.19E-38 | 2.03E-36 |
| ACAD11 | 3.211125 | 1.40E-38 | 2.34E-36 |
| ZNF337 | 2.757743 | 1.40E-38 | 2.34E-36 |
| CDK3 | 3.084775 | 1.43E-38 | 2.36E-36 |
| TRPV1 | 2.250695 | 1.47E-38 | 2.41E-36 |
| PLA2G4B | 3.529791 | 1.48E-38 | 2.42E-36 |
| SCN7A | -2.42723 | 1.51E-38 | 2.43E-36 |
| NHEJ1 | 2.646663 | 1.84E-38 | 2.94E-36 |
| MASP1 | -3.02473 | 1.91E-38 | 3.03E-36 |
| INMT | -2.8465 | 2.65E-38 | 4.17E-36 |
| RPL36A | 3.554017 | 2.91E-38 | 4.55E-36 |
| MAN2C1 | 2.660823 | 3.03E-38 | 4.68E-36 |
| JPH2 | -3.14389 | 3.25E-38 | 4.99E-36 |
| TM9SF1 | 2.42676 | 4.05E-38 | 6.17E-36 |
| LIN37 | 2.323879 | 4.52E-38 | 6.82E-36 |
| ASB2 | -2.47926 | 5.82E-38 | 8.66E-36 |
| PYURF | 3.711694 | 8.88E-38 | 1.29E-35 |
| NPEPL1 | 2.880218 | 1.05E-37 | 1.51E-35 |
| ZNF20 | 2.023081 | 1.25E-37 | 1.79E-35 |
| IPO4 | 2.777993 | 1.49E-37 | 2.12E-35 |
| CHKB | 3.43436 | 1.53E-37 | 2.15E-35 |
| TUBA8 | 2.158188 | 1.56E-37 | 2.18E-35 |
| MGAT2 | 2.936366 | 1.58E-37 | 2.19E-35 |
| OVCA2 | 3.223572 | 2.10E-37 | 2.87E-35 |
| RGL4 | 2.5266 | 2.15E-37 | 2.91E-35 |
| SMYD3 | 2.505311 | 4.27E-37 | 5.68E-35 |
| MRPS17 | 2.008717 | 4.65E-37 | 6.13E-35 |
| H3F3A | 2.554008 | 5.57E-37 | 7.25E-35 |
| ARHGAP8 | 3.063883 | 5.84E-37 | 7.55E-35 |
| DDX39B | 2.842157 | 7.70E-37 | 9.81E-35 |
| C12orf45 | -2.67242 | 8.32E-37 | 1.05E-34 |
| LAT | 3.327525 | 8.54E-37 | 1.07E-34 |
| MTG1 | 3.082769 | 9.24E-37 | 1.15E-34 |
| MYOCD | -2.4473 | 1.22E-36 | 1.49E-34 |
| BBS1 | 2.929 | 1.54E-36 | 1.88E-34 |
| PDXP | 2.837047 | 1.83E-36 | 2.17E-34 |
| UBD | -3.38536 | 2.16E-36 | 2.55E-34 |
| ATP5O | 2.238296 | 2.75E-36 | 3.23E-34 |
| SYNM | -3.58482 | 2.89E-36 | 3.36E-34 |
| RPL17 | 4.227601 | 3.64E-36 | 4.21E-34 |
| TRAPPC5 | 2.846293 | 3.85E-36 | 4.42E-34 |
| KCNMB1 | -2.92572 | 4.26E-36 | 4.86E-34 |
| CYB5D1 | 2.177932 | 5.22E-36 | 5.93E-34 |
| TICAM2 | 2.632462 | 5.92E-36 | 6.68E-34 |
| MKX | -2.66697 | 6.67E-36 | 7.44E-34 |
| CCDC117 | 2.664794 | 1.08E-35 | 1.20E-33 |
| PAGE4 | -3.84998 | 1.12E-35 | 1.24E-33 |
| RP11-638I8.1 | -2.59093 | 1.39E-35 | 1.52E-33 |
| HYPK | 2.915352 | 1.51E-35 | 1.64E-33 |
| BCKDHA | 2.487282 | 2.51E-35 | 2.70E-33 |
| TNFRSF6B | 3.218964 | 2.73E-35 | 2.93E-33 |
| ADCY5 | -2.54324 | 2.93E-35 | 3.11E-33 |
| SPP1 | 4.460282 | 2.95E-35 | 3.12E-33 |
| HIST1H2AD | -3.66671 | 3.41E-35 | 3.59E-33 |
| SAPCD1 | 3.06811 | 4.69E-35 | 4.90E-33 |
| BGLAP | 5.130559 | 7.54E-35 | 7.85E-33 |
| ALX3 | 2.118446 | 9.71E-35 | 1.00E-32 |
| SRXN1 | 3.416688 | 1.94E-34 | 1.98E-32 |
| SAP25 | 2.99639 | 2.20E-34 | 2.22E-32 |
| PTPRCAP | 3.797089 | 2.76E-34 | 2.78E-32 |
| KREMEN1 | 3.273228 | 3.38E-34 | 3.38E-32 |
| CSDC2 | -2.38898 | 4.71E-34 | 4.66E-32 |
| EGFL8 | 3.011094 | 4.87E-34 | 4.80E-32 |
| ZGPAT | 2.167478 | 6.73E-34 | 6.50E-32 |
| C8orf76 | 2.369938 | 6.99E-34 | 6.71E-32 |
| SLC31A2 | 2.787031 | 7.59E-34 | 7.23E-32 |
| ALKBH6 | 2.488094 | 7.92E-34 | 7.50E-32 |
| CHRDL1 | -4.17777 | 8.82E-34 | 8.30E-32 |
| HBA2 | 6.096543 | 9.57E-34 | 8.97E-32 |
| RPL30 | 2.290328 | 1.43E-33 | 1.33E-31 |
| ACY1 | 2.819044 | 1.59E-33 | 1.48E-31 |
| MYL9 | -3.74534 | 1.71E-33 | 1.57E-31 |
| MTHFS | 2.424671 | 1.95E-33 | 1.79E-31 |
| CORO7 | 2.346362 | 2.40E-33 | 2.18E-31 |
| ECSCR | 2.721305 | 3.06E-33 | 2.77E-31 |
| INO80B | 2.459647 | 3.29E-33 | 2.96E-31 |
| NR2F1 | -2.91686 | 5.36E-33 | 4.76E-31 |
| NDUFV2 | 2.626314 | 6.18E-33 | 5.46E-31 |
| MRPS24 | 3.147087 | 6.35E-33 | 5.59E-31 |
| MEMO1 | 2.200496 | 7.42E-33 | 6.46E-31 |
| CPT1B | 3.026453 | 7.68E-33 | 6.66E-31 |
| MSH5 | 2.649917 | 8.12E-33 | 7.00E-31 |
| ZNRF3 | 2.547395 | 9.29E-33 | 7.91E-31 |
| ALDH1A2 | -2.34895 | 1.19E-32 | 1.01E-30 |
| 4-Sep | 2.534479 | 1.25E-32 | 1.05E-30 |
| RPS10 | 2.035404 | 1.32E-32 | 1.10E-30 |
| FEM1A | 2.336714 | 1.40E-32 | 1.16E-30 |
| PPAN | 2.240318 | 1.58E-32 | 1.31E-30 |
| SP110 | 2.555987 | 1.89E-32 | 1.56E-30 |
| TCEAL2 | -3.46969 | 2.30E-32 | 1.88E-30 |
| MEIS2 | -2.42354 | 2.49E-32 | 2.02E-30 |
| GIMAP5 | 2.84961 | 2.61E-32 | 2.10E-30 |
| CKLF | 2.379848 | 2.67E-32 | 2.15E-30 |
| ISL1 | -2.47966 | 2.91E-32 | 2.33E-30 |
| FHL2 | -2.57425 | 3.17E-32 | 2.53E-30 |
| GAPDHS | 2.775974 | 3.33E-32 | 2.64E-30 |
| SCHIP1 | 2.382355 | 3.76E-32 | 2.97E-30 |
| EGLN2 | 2.027717 | 4.20E-32 | 3.31E-30 |
| UQCR11 | 3.079026 | 4.80E-32 | 3.76E-30 |
| ABCB6 | 2.390117 | 4.86E-32 | 3.79E-30 |
| SPOCK3 | -3.81207 | 5.76E-32 | 4.48E-30 |
| SRD5A2 | -2.9817 | 6.26E-32 | 4.84E-30 |
| FAM46B | -2.58861 | 8.71E-32 | 6.66E-30 |
| C7 | -3.78895 | 1.10E-31 | 8.35E-30 |
| CHMP4A | 3.12676 | 1.36E-31 | 1.02E-29 |
| KRT5 | -5.58918 | 1.36E-31 | 1.02E-29 |
| SLC24A3 | -2.5034 | 1.52E-31 | 1.12E-29 |
| RANGRF | -2.76506 | 1.72E-31 | 1.27E-29 |
| CDH15 | 3.050716 | 1.83E-31 | 1.34E-29 |
| EEF1D | 2.442982 | 2.04E-31 | 1.49E-29 |
| PHYHIP | -2.01271 | 2.09E-31 | 1.53E-29 |
| C9orf129 | 3.301114 | 2.43E-31 | 1.76E-29 |
| IRF9 | 3.303146 | 2.58E-31 | 1.86E-29 |
| COX6A1 | 2.036281 | 2.81E-31 | 2.02E-29 |
| SFRP1 | -3.70898 | 3.27E-31 | 2.34E-29 |
| DLEU1 | 2.322125 | 3.70E-31 | 2.63E-29 |
| TMEM200B | -2.19231 | 3.86E-31 | 2.74E-29 |
| TNFAIP6 | 2.782979 | 3.91E-31 | 2.75E-29 |
| VASH1 | 2.094732 | 4.82E-31 | 3.38E-29 |
| ILK | 2.450867 | 4.98E-31 | 3.48E-29 |
| CCL19 | -3.39375 | 5.16E-31 | 3.59E-29 |
| LRRC15 | 3.345301 | 7.01E-31 | 4.86E-29 |
| TACC3 | 2.610351 | 7.48E-31 | 5.17E-29 |
| LRRC75A | 2.16283 | 9.48E-31 | 6.48E-29 |
| SLC7A3 | -2.4656 | 1.09E-30 | 7.38E-29 |
| GALNT4 | 2.215465 | 1.12E-30 | 7.53E-29 |
| SULT1A1 | 2.233313 | 1.26E-30 | 8.49E-29 |
| PGF | 2.911556 | 1.37E-30 | 9.18E-29 |
| CD36 | 3.389195 | 1.50E-30 | 9.98E-29 |
| FBF1 | 2.0589 | 1.50E-30 | 9.98E-29 |
| COL4A6 | -2.24713 | 1.56E-30 | 1.03E-28 |
| 1-Mar | 2.236011 | 1.65E-30 | 1.08E-28 |
| LIME1 | 2.644447 | 1.74E-30 | 1.14E-28 |
| RPP21 | 2.253076 | 2.16E-30 | 1.40E-28 |
| SORBS1 | -2.88551 | 2.63E-30 | 1.68E-28 |
| MAOB | -4.31892 | 2.63E-30 | 1.68E-28 |
| LRRC8E | 2.024106 | 2.94E-30 | 1.84E-28 |
| ITGA10 | 2.680816 | 3.08E-30 | 1.92E-28 |
| C2orf40 | -3.14508 | 4.33E-30 | 2.67E-28 |
| TWIST2 | -2.23313 | 5.38E-30 | 3.27E-28 |
| DCDC2B | 2.277764 | 6.05E-30 | 3.66E-28 |
| CLIC6 | -2.10644 | 6.46E-30 | 3.89E-28 |
| PDZRN4 | -2.14102 | 9.88E-30 | 5.86E-28 |
| MDP1 | 2.225269 | 1.08E-29 | 6.37E-28 |
| APOC2 | 2.808213 | 1.15E-29 | 6.80E-28 |
| MUSTN1 | 2.666291 | 1.75E-29 | 1.02E-27 |
| HLF | -2.2766 | 1.82E-29 | 1.06E-27 |
| PHOSPHO1 | 2.830015 | 1.91E-29 | 1.11E-27 |
| MRVI1 | -2.45729 | 2.53E-29 | 1.45E-27 |
| CDRT4 | 2.129213 | 3.61E-29 | 2.05E-27 |
| STMN1 | 2.224206 | 3.67E-29 | 2.08E-27 |
| MMP9 | 4.266006 | 5.13E-29 | 2.87E-27 |
| MMP11 | 2.780793 | 5.98E-29 | 3.33E-27 |
| ACKR1 | -3.51968 | 1.15E-28 | 6.26E-27 |
| PTH1R | 3.368212 | 1.31E-28 | 7.12E-27 |
| KLK10 | -2.44299 | 1.52E-28 | 8.19E-27 |
| FAM107A | -2.8999 | 1.63E-28 | 8.77E-27 |
| IFITM5 | 3.854363 | 1.73E-28 | 9.22E-27 |
| TAGLN | -3.21725 | 1.90E-28 | 9.99E-27 |
| FAIM2 | -2.40034 | 2.01E-28 | 1.06E-26 |
| HOXD10 | -2.21543 | 2.65E-28 | 1.37E-26 |
| CPN2 | 2.077438 | 3.00E-28 | 1.55E-26 |
| RDH5 | 2.111545 | 3.98E-28 | 2.04E-26 |
| ADORA2A | 2.265964 | 5.05E-28 | 2.56E-26 |
| MCMDC2 | 2.401582 | 6.47E-28 | 3.25E-26 |
| SLC25A6 | 2.066562 | 1.15E-27 | 5.69E-26 |
| SEC14L2 | -2.32662 | 1.45E-27 | 7.09E-26 |
| CCL21 | -3.24991 | 1.72E-27 | 8.33E-26 |
| FOXM1 | 2.220085 | 1.94E-27 | 9.34E-26 |
| WBP1 | 2.103023 | 2.15E-27 | 1.02E-25 |
| C1QTNF6 | 2.420382 | 2.29E-27 | 1.08E-25 |
| PRICKLE4 | 2.164802 | 2.78E-27 | 1.30E-25 |
| AOC3 | -2.27842 | 2.81E-27 | 1.31E-25 |
| CHAD | 4.471504 | 2.99E-27 | 1.39E-25 |
| EFEMP1 | -3.31144 | 3.42E-27 | 1.58E-25 |
| HSPB8 | -3.71557 | 4.69E-27 | 2.13E-25 |
| C18orf32 | 2.012233 | 4.92E-27 | 2.22E-25 |
| HSD11B1 | -2.26913 | 5.32E-27 | 2.39E-25 |
| OLFML2B | 2.800949 | 5.69E-27 | 2.55E-25 |
| TUBB3 | 3.036281 | 5.85E-27 | 2.62E-25 |
| LAMA2 | -2.1028 | 7.81E-27 | 3.45E-25 |
| PAM16 | 2.051936 | 9.53E-27 | 4.16E-25 |
| SP7 | 2.334851 | 1.01E-26 | 4.42E-25 |
| PCSK7 | 2.083475 | 1.06E-26 | 4.58E-25 |
| PDPN | -2.08447 | 1.31E-26 | 5.66E-25 |
| HDAC10 | 2.284512 | 1.61E-26 | 6.88E-25 |
| C20orf24 | 2.002537 | 1.67E-26 | 7.07E-25 |
| PLK1 | 2.49239 | 1.79E-26 | 7.57E-25 |
| PLN | -2.458 | 1.82E-26 | 7.66E-25 |
| A1BG | 2.239268 | 2.05E-26 | 8.54E-25 |
| AOX1 | -2.4908 | 2.63E-26 | 1.09E-24 |
| CHRDL2 | -2.33279 | 2.90E-26 | 1.20E-24 |
| MSMB | -5.75314 | 3.39E-26 | 1.39E-24 |
| GATA3 | -2.41894 | 3.41E-26 | 1.40E-24 |
| NDUFA13 | 2.154489 | 4.19E-26 | 1.70E-24 |
| CA2 | 3.508598 | 4.76E-26 | 1.92E-24 |
| SH3BP5 | 2.065095 | 4.79E-26 | 1.92E-24 |
| COL11A1 | 3.295672 | 5.24E-26 | 2.09E-24 |
| SGCA | -2.29463 | 6.07E-26 | 2.42E-24 |
| VIM | 2.308156 | 7.35E-26 | 2.89E-24 |
| SELP | -2.26684 | 8.30E-26 | 3.24E-24 |
| PAMR1 | -2.23072 | 1.07E-25 | 4.14E-24 |
| NEXN | -2.45578 | 1.36E-25 | 5.22E-24 |
| RAB43 | 2.034565 | 1.96E-25 | 7.43E-24 |
| RAB24 | 2.096331 | 2.69E-25 | 1.00E-23 |
| AS3MT | 2.090642 | 2.78E-25 | 1.03E-23 |
| ERVK3-1 | 2.190793 | 2.88E-25 | 1.06E-23 |
| FMO3 | 2.371115 | 5.55E-25 | 2.00E-23 |
| HMOX1 | 2.621409 | 6.76E-25 | 2.42E-23 |
| RPRM | -2.31523 | 1.21E-24 | 4.27E-23 |
| PTTG1 | 2.443478 | 1.23E-24 | 4.32E-23 |
| TNS4 | -3.28923 | 1.95E-24 | 6.75E-23 |
| TIMP3 | 3.34212 | 2.26E-24 | 7.75E-23 |
| SCGB1A1 | -4.41529 | 2.74E-24 | 9.38E-23 |
| JPH4 | -2.25721 | 3.11E-24 | 1.05E-22 |
| PNP | 2.08333 | 3.51E-24 | 1.19E-22 |
| KCND3 | -2.63262 | 3.65E-24 | 1.23E-22 |
| SFRP2 | -3.89537 | 5.47E-24 | 1.82E-22 |
| PRELP | -3.11442 | 5.70E-24 | 1.89E-22 |
| MYLK | -2.67532 | 6.56E-24 | 2.15E-22 |
| COL5A3 | 2.055477 | 6.97E-24 | 2.27E-22 |
| KLRK1 | 2.364299 | 7.26E-24 | 2.37E-22 |
| RRM2 | 2.249581 | 8.41E-24 | 2.73E-22 |
| KRT15 | -4.11912 | 9.08E-24 | 2.92E-22 |
| IL33 | -2.34238 | 1.41E-23 | 4.44E-22 |
| SPARCL1 | -2.62295 | 1.41E-23 | 4.44E-22 |
| LTBP4 | -2.27641 | 1.67E-23 | 5.19E-22 |
| GPR87 | -2.43452 | 1.68E-23 | 5.22E-22 |
| ORC6 | 2.094368 | 1.76E-23 | 5.43E-22 |
| APLNR | 2.486747 | 2.58E-23 | 7.86E-22 |
| TPGS1 | 2.113526 | 2.70E-23 | 8.16E-22 |
| MEPE | 2.95713 | 4.24E-23 | 1.27E-21 |
| CD27 | 2.327726 | 4.41E-23 | 1.31E-21 |
| CES1 | -2.46761 | 4.57E-23 | 1.36E-21 |
| CTF1 | -2.07696 | 5.50E-23 | 1.62E-21 |
| DMP1 | 2.446614 | 6.65E-23 | 1.95E-21 |
| ITGB3 | 2.186791 | 7.83E-23 | 2.28E-21 |
| HBA1 | 4.594601 | 1.83E-22 | 5.15E-21 |
| GCNT2 | -2.21798 | 1.88E-22 | 5.27E-21 |
| TRIM29 | -3.34395 | 1.95E-22 | 5.42E-21 |
| ITGB3BP | 2.092126 | 3.06E-22 | 8.38E-21 |
| CCDC78 | 2.277915 | 3.61E-22 | 9.80E-21 |
| IGFBP6 | -2.44322 | 3.78E-22 | 1.02E-20 |
| ELOVL7 | -2.29372 | 4.17E-22 | 1.12E-20 |
| FIBIN | -2.40436 | 4.60E-22 | 1.23E-20 |
| HSPB6 | -3.1436 | 5.46E-22 | 1.45E-20 |
| COL11A2 | 2.118898 | 6.34E-22 | 1.66E-20 |
| DUSP1 | -2.9793 | 6.43E-22 | 1.68E-20 |
| PODN | -2.33372 | 6.98E-22 | 1.82E-20 |
| HAGHL | 2.316678 | 7.77E-22 | 2.01E-20 |
| FLRT3 | -2.12984 | 1.03E-21 | 2.61E-20 |
| CDK1 | 2.514005 | 1.05E-21 | 2.66E-20 |
| KRT14 | -3.75384 | 1.18E-21 | 2.99E-20 |
| ALPL | 3.30162 | 1.28E-21 | 3.21E-20 |
| PARM1 | -2.72656 | 1.89E-21 | 4.62E-20 |
| SPON1 | -2.30053 | 2.07E-21 | 5.03E-20 |
| PRDM8 | -2.26831 | 2.20E-21 | 5.32E-20 |
| FNDC1 | 2.764372 | 2.77E-21 | 6.63E-20 |
| IFI27 | 2.720542 | 2.82E-21 | 6.75E-20 |
| UPK3BL | 2.898138 | 3.49E-21 | 8.26E-20 |
| ITGA8 | -2.24662 | 3.56E-21 | 8.42E-20 |
| COL1A1 | 3.290336 | 4.54E-21 | 1.06E-19 |
| MYOC | -2.01224 | 4.58E-21 | 1.07E-19 |
| COL1A2 | 2.919818 | 5.31E-21 | 1.23E-19 |
| CAPN6 | -2.24328 | 6.78E-21 | 1.55E-19 |
| TROAP | 2.006494 | 8.32E-21 | 1.89E-19 |
| HPSE2 | -2.59864 | 9.11E-21 | 2.06E-19 |
| NPIPA1 | 2.348762 | 9.17E-21 | 2.07E-19 |
| THBS4 | -3.05911 | 1.03E-20 | 2.31E-19 |
| TINCR | -2.11652 | 1.13E-20 | 2.53E-19 |
| COL5A2 | 2.489053 | 1.33E-20 | 2.93E-19 |
| GABRP | -2.46629 | 1.36E-20 | 2.98E-19 |
| TP63 | -2.43966 | 1.37E-20 | 3.01E-19 |
| KRT17 | -3.84172 | 1.45E-20 | 3.17E-19 |
| XAF1 | 2.550273 | 1.46E-20 | 3.17E-19 |
| CTSK | 2.7002 | 1.60E-20 | 3.47E-19 |
| COL17A1 | -2.51851 | 1.66E-20 | 3.58E-19 |
| KIF20A | 2.00912 | 2.05E-20 | 4.37E-19 |
| DSC3 | -2.64009 | 2.32E-20 | 4.95E-19 |
| TRPM8 | -4.59242 | 2.55E-20 | 5.40E-19 |
| GPC3 | -2.05655 | 3.06E-20 | 6.40E-19 |
| HOXD13 | -2.27763 | 3.35E-20 | 6.98E-19 |
| LDB3 | -2.21677 | 3.41E-20 | 7.10E-19 |
| ACPP | -3.28221 | 3.44E-20 | 7.16E-19 |
| LINC00890 | -2.6991 | 3.66E-20 | 7.61E-19 |
| FAM189A2 | -2.81462 | 3.78E-20 | 7.85E-19 |
| EMC6 | 2.115436 | 3.99E-20 | 8.24E-19 |
| INSC | 2.037232 | 6.28E-20 | 1.26E-18 |
| PLOD2 | 2.168035 | 7.82E-20 | 1.55E-18 |
| INAFM2 | -2.05509 | 8.88E-20 | 1.74E-18 |
| KLF5 | -2.30341 | 9.06E-20 | 1.78E-18 |
| CXCL17 | -2.702 | 9.41E-20 | 1.84E-18 |
| SLC14A1 | -2.92357 | 1.07E-19 | 2.08E-18 |
| TPM2 | -2.32525 | 1.09E-19 | 2.12E-18 |
| FN1 | 2.242745 | 1.64E-19 | 3.13E-18 |
| TACSTD2 | -2.08131 | 1.70E-19 | 3.24E-18 |
| PRSS35 | 2.79101 | 2.55E-19 | 4.76E-18 |
| APELA | -2.03368 | 2.85E-19 | 5.30E-18 |
| OGN | -2.43626 | 2.85E-19 | 5.31E-18 |
| C1orf115 | -2.39302 | 3.91E-19 | 7.20E-18 |
| ZFP36 | -2.97354 | 4.52E-19 | 8.24E-18 |
| ISLR | -2.12273 | 5.32E-19 | 9.64E-18 |
| HSPB7 | -2.06786 | 5.97E-19 | 1.08E-17 |
| PCOLCE | 2.30758 | 8.92E-19 | 1.57E-17 |
| HBM | 3.557602 | 9.29E-19 | 1.63E-17 |
| NDUFA4L2 | 2.080453 | 9.94E-19 | 1.74E-17 |
| SOD3 | -2.32637 | 1.16E-18 | 2.01E-17 |
| CPLX3 | 3.298383 | 1.34E-18 | 2.31E-17 |
| ST6GALNAC1 | -3.01371 | 1.43E-18 | 2.45E-17 |
| PTX3 | 2.591106 | 1.50E-18 | 2.57E-17 |
| DHCR24 | -2.70382 | 1.69E-18 | 2.88E-17 |
| SULT2B1 | -2.41562 | 1.85E-18 | 3.14E-17 |
| ATP1A2 | -2.42988 | 2.13E-18 | 3.59E-17 |
| FBLN5 | -2.19645 | 2.16E-18 | 3.64E-17 |
| PROK1 | -2.30551 | 2.32E-18 | 3.88E-17 |
| ESM1 | 2.492172 | 3.34E-18 | 5.49E-17 |
| PF4 | 3.133844 | 4.23E-18 | 6.86E-17 |
| PHYHD1 | -2.33117 | 4.25E-18 | 6.89E-17 |
| NUF2 | 2.217587 | 5.05E-18 | 8.08E-17 |
| SERPINF2 | -2.0752 | 5.22E-18 | 8.34E-17 |
| CACNG4 | -3.0037 | 5.27E-18 | 8.43E-17 |
| PABPC1L2B | -2.20517 | 7.24E-18 | 1.14E-16 |
| PKP1 | -3.34274 | 7.24E-18 | 1.14E-16 |
| AURKB | 2.072035 | 7.66E-18 | 1.20E-16 |
| FABP4 | 3.077154 | 9.02E-18 | 1.40E-16 |
| SLC4A4 | -3.35945 | 9.41E-18 | 1.46E-16 |
| ALAS2 | 3.002106 | 9.98E-18 | 1.54E-16 |
| PTGS2 | -2.51036 | 1.49E-17 | 2.25E-16 |
| PPBP | 3.196811 | 1.61E-17 | 2.41E-16 |
| PI16 | -2.63546 | 1.80E-17 | 2.68E-16 |
| TOP2A | 2.181848 | 1.98E-17 | 2.93E-16 |
| NUSAP1 | 2.01958 | 2.27E-17 | 3.33E-16 |
| ALDH1A3 | -2.08948 | 2.28E-17 | 3.35E-16 |
| MMP13 | 2.550166 | 2.37E-17 | 3.48E-16 |
| SDPR | -2.07274 | 2.43E-17 | 3.56E-16 |
| LAMB3 | -2.70739 | 2.46E-17 | 3.60E-16 |
| CTGF | -2.33053 | 2.50E-17 | 3.65E-16 |
| CHRM1 | -2.72465 | 2.57E-17 | 3.73E-16 |
| S100A2 | -2.80601 | 3.06E-17 | 4.40E-16 |
| MYL4 | 2.279998 | 3.52E-17 | 4.99E-16 |
| ANO7 | -3.10651 | 6.90E-17 | 9.40E-16 |
| ITGB4 | -2.09213 | 7.51E-17 | 1.02E-15 |
| ACTA2 | -2.35845 | 8.04E-17 | 1.09E-15 |
| CSF3R | 2.348761 | 1.43E-16 | 1.88E-15 |
| DPP4 | -3.00096 | 1.68E-16 | 2.19E-15 |
| KRT7 | -3.03435 | 2.14E-16 | 2.76E-15 |
| STEAP4 | -2.53608 | 3.65E-16 | 4.57E-15 |
| TMEM30B | -2.10683 | 4.48E-16 | 5.59E-15 |
| EGR2 | -2.20522 | 4.60E-16 | 5.73E-15 |
| AHSP | 3.356843 | 6.16E-16 | 7.51E-15 |
| IGFBP5 | -2.27995 | 6.45E-16 | 7.82E-15 |
| FMOD | -2.5871 | 6.58E-16 | 7.96E-15 |
| TSPAN1 | -2.45645 | 7.19E-16 | 8.65E-15 |
| GLB1L3 | -2.38075 | 7.59E-16 | 9.09E-15 |
| PTPRN2 | -2.64453 | 7.64E-16 | 9.14E-15 |
| HBD | 3.496998 | 9.67E-16 | 1.14E-14 |
| S100A14 | -2.572 | 9.69E-16 | 1.14E-14 |
| CECR6 | -2.03345 | 1.20E-15 | 1.40E-14 |
| SLC22A3 | -2.66818 | 1.30E-15 | 1.51E-14 |
| S100A8 | 3.771622 | 3.27E-15 | 3.65E-14 |
| SLC15A2 | -2.22644 | 3.87E-15 | 4.29E-14 |
| ENTPD5 | -2.02028 | 4.12E-15 | 4.54E-14 |
| CYP4B1 | -2.14567 | 4.76E-15 | 5.20E-14 |
| CPZ | 2.108749 | 5.14E-15 | 5.60E-14 |
| CHRNA2 | -3.00754 | 6.09E-15 | 6.58E-14 |
| KLK11 | -3.85141 | 7.48E-15 | 7.95E-14 |
| SLC4A1 | 2.729142 | 7.56E-15 | 8.03E-14 |
| HK3 | 2.001506 | 8.12E-15 | 8.58E-14 |
| SPDEF | -2.24159 | 8.90E-15 | 9.34E-14 |
| FEV | -2.57711 | 9.27E-15 | 9.71E-14 |
| SP8 | -2.02003 | 1.38E-14 | 1.41E-13 |
| CXCL11 | -2.11353 | 1.39E-14 | 1.42E-13 |
| SLC7A4 | -2.00813 | 1.44E-14 | 1.46E-13 |
| APOE | 2.180714 | 1.59E-14 | 1.61E-13 |
| SLC6A14 | -2.03899 | 1.85E-14 | 1.86E-13 |
| MIF | 2.067405 | 2.20E-14 | 2.18E-13 |
| CD38 | -2.61191 | 2.39E-14 | 2.36E-13 |
| ADRB1 | -2.36615 | 3.56E-14 | 3.42E-13 |
| NR4A3 | -2.00828 | 3.97E-14 | 3.77E-13 |
| FOS | -3.30853 | 4.64E-14 | 4.36E-13 |
| CYR61 | -2.19253 | 4.65E-14 | 4.37E-13 |
| APOF | -2.13444 | 4.76E-14 | 4.46E-13 |
| SLC26A4 | -2.1887 | 4.91E-14 | 4.57E-13 |
| VCAN | 2.001168 | 5.06E-14 | 4.70E-13 |
| CREB3L1 | -2.12673 | 6.43E-14 | 5.90E-13 |
| ABCC4 | -2.52581 | 1.06E-13 | 9.53E-13 |
| CITED1 | -2.16115 | 1.44E-13 | 1.27E-12 |
| PEBP4 | -2.7871 | 1.68E-13 | 1.47E-12 |
| ACTC1 | -2.38559 | 1.68E-13 | 1.47E-12 |
| LPAR3 | -2.28078 | 2.03E-13 | 1.75E-12 |
| EGR1 | -2.93184 | 2.14E-13 | 1.83E-12 |
| LAMC2 | -2.15796 | 2.75E-13 | 2.33E-12 |
| SAA1 | -2.15916 | 3.28E-13 | 2.76E-12 |
| CLDN1 | -2.28799 | 3.92E-13 | 3.27E-12 |
| CUX2 | -2.13973 | 3.94E-13 | 3.28E-12 |
| HBG2 | 2.281379 | 4.25E-13 | 3.52E-12 |
| HSD17B6 | -3.01202 | 4.56E-13 | 3.76E-12 |
| GOLM1 | -2.04265 | 5.30E-13 | 4.34E-12 |
| SLC45A3 | -2.65913 | 5.50E-13 | 4.49E-12 |
| NR4A1 | -2.17239 | 5.59E-13 | 4.56E-12 |
| RASD1 | -2.00156 | 8.33E-13 | 6.63E-12 |
| ACP5 | 2.161714 | 8.38E-13 | 6.66E-12 |
| CA1 | 2.121897 | 8.64E-13 | 6.86E-12 |
| TMSB15A | -2.51161 | 9.14E-13 | 7.23E-12 |
| MMP7 | -3.38279 | 9.35E-13 | 7.38E-12 |
| F3 | -2.73593 | 9.57E-13 | 7.55E-12 |
| AKR1C3 | 2.078009 | 1.07E-12 | 8.39E-12 |
| CLDN8 | -2.67954 | 1.17E-12 | 9.13E-12 |
| CAMP | 2.546253 | 1.37E-12 | 1.05E-11 |
| CHGA | -2.73088 | 1.51E-12 | 1.15E-11 |
| S100A12 | 2.922393 | 2.06E-12 | 1.54E-11 |
| CLC | 2.348995 | 2.14E-12 | 1.60E-11 |
| KRT23 | -2.10244 | 2.49E-12 | 1.84E-11 |
| SFN | -2.69104 | 3.72E-12 | 2.67E-11 |
| IGLL5 | 3.229453 | 3.72E-12 | 2.67E-11 |
| PRG2 | 2.432464 | 6.75E-12 | 4.70E-11 |
| DEFB1 | -2.57071 | 9.68E-12 | 6.60E-11 |
| ALOX15B | -4.01392 | 1.10E-11 | 7.49E-11 |
| BPIFB2 | -2.28999 | 1.14E-11 | 7.71E-11 |
| OLFM4 | -3.51928 | 1.48E-11 | 9.87E-11 |
| LINC01207 | -2.42238 | 1.53E-11 | 1.01E-10 |
| GREB1 | -2.03918 | 1.74E-11 | 1.15E-10 |
| OR51E2 | -4.03256 | 2.05E-11 | 1.34E-10 |
| SOCS3 | -2.04199 | 3.57E-11 | 2.27E-10 |
| C2orf72 | -2.36667 | 4.53E-11 | 2.84E-10 |
| PTN | -2.09527 | 5.06E-11 | 3.14E-10 |
| SPOCK1 | -2.40628 | 5.78E-11 | 3.56E-10 |
| KLK3 | -2.77072 | 8.42E-11 | 5.08E-10 |
| PLA1A | -2.38785 | 1.36E-10 | 7.91E-10 |
| FOSB | -3.01281 | 1.48E-10 | 8.60E-10 |
| DEFA3 | 2.15533 | 2.01E-10 | 1.14E-09 |
| DNASE2B | -2.0036 | 3.24E-10 | 1.79E-09 |
| VSTM2L | -2.33564 | 3.52E-10 | 1.93E-09 |
| PIGR | -3.07404 | 5.18E-10 | 2.79E-09 |
| AZU1 | 2.327805 | 7.12E-10 | 3.77E-09 |
| VSIG2 | -2.23131 | 8.55E-10 | 4.46E-09 |
| KRT13 | -2.49578 | 1.85E-09 | 9.20E-09 |
| CEACAM20 | -2.04941 | 2.35E-09 | 1.15E-08 |
| S100A9 | 2.661849 | 2.79E-09 | 1.35E-08 |
| DEFA4 | 2.278073 | 3.12E-09 | 1.50E-08 |
| MPO | 2.252925 | 8.97E-09 | 4.09E-08 |
| NEFH | -3.51316 | 9.21E-09 | 4.19E-08 |
| TFF1 | -2.33054 | 1.02E-08 | 4.61E-08 |
| PGC | -2.61046 | 1.37E-08 | 6.08E-08 |
| AZGP1 | -3.07432 | 1.72E-08 | 7.51E-08 |
| GSTA1 | -2.13442 | 2.52E-08 | 1.07E-07 |
| PRTN3 | 2.218278 | 2.54E-08 | 1.08E-07 |
| PI15 | -2.30913 | 4.14E-08 | 1.71E-07 |
| PLA2G2A | -2.89724 | 5.10E-08 | 2.08E-07 |
| SERPINA3 | 2.084001 | 5.24E-08 | 2.13E-07 |
| UGT2B15 | 2.283554 | 5.69E-08 | 2.31E-07 |
| NPY | -4.39361 | 6.04E-08 | 2.44E-07 |
| LTF | -2.72068 | 1.63E-07 | 6.17E-07 |
| MYBPC1 | -2.09022 | 1.81E-07 | 6.81E-07 |
| TFF3 | -2.28263 | 3.22E-07 | 1.17E-06 |
| OR51E1 | -2.17343 | 1.06E-06 | 3.55E-06 |
| RLN1 | -2.54691 | 3.90E-06 | 1.20E-05 |
| CD177 | -2.05826 | 1.15E-05 | 3.29E-05 |
| TGM4 | -2.39188 | 2.08E-05 | 5.74E-05 |
